# Anti-TIGIT antibody improves PD-L1 blockade through myeloid and T_reg_ cells

**DOI:** 10.1038/s41586-024-07121-9

**Published:** 2024-02-28

**Authors:** Xiangnan Guan, Ruozhen Hu, Yoonha Choi, Shyam Srivats, Barzin Y. Nabet, John Silva, Lisa McGinnis, Robert Hendricks, Katherine Nutsch, Karl L. Banta, Ellen Duong, Alexis Dunkle, Patrick S. Chang, Chia-Jung Han, Stephanie Mittman, Nandini Molden, Pallavi Daggumati, Wendy Connolly, Melissa Johnson, Delvys Rodriguez Abreu, Byoung Chul Cho, Antoine Italiano, Ignacio Gil-Bazo, Enriqueta Felip, Ira Mellman, Sanjeev Mariathasan, David S. Shames, Raymond Meng, Eugene Y. Chiang, Robert J. Johnston, Namrata S. Patil

**Affiliations:** 1 Genentech Inc., South San Francisco, CA USA; 2 Sarah Cannon Research Institute/Tennessee Oncology, PLLC, Nashville, TN USA; 3 Hospital Universitario Insular de Gran Canaria, Las Palmas, Spain; 4 Yonsei Cancer Centre, Yonsei University College of Medicine, Seoul, South Korea; 5 Institut Bergonie CLCC Bordeaux, Bordeaux, France; 6 Faculty of Medicine, University of Bordeaux, Bordeaux, France; 7 Clínica Universidad de Navarra, CIMA Universidad de Navarra Pamplona, Pamplona, Spain; 8 Vall d’Hebron Institute of Oncology (VHIO), Barcelona, Spain

**Keywords:** Non-small-cell lung cancer, Biomarkers

## Abstract

Tiragolumab, an anti-TIGIT antibody with an active IgG1κ Fc, demonstrated improved outcomes in the phase 2 CITYSCAPE trial (ClinicalTrials.gov: NCT03563716) when combined with atezolizumab (anti-PD-L1) versus atezolizumab alone^1^. However, there remains little consensus on the mechanism(s) of response with this combination^2^. Here we find that a high baseline of intratumoural macrophages and regulatory T cells is associated with better outcomes in patients treated with atezolizumab plus tiragolumab but not with atezolizumab alone. Serum sample analysis revealed that macrophage activation is associated with a clinical benefit in patients who received the combination treatment. In mouse tumour models, tiragolumab surrogate antibodies inflamed tumour-associated macrophages, monocytes and dendritic cells through Fcγ receptors (FcγR), in turn driving anti-tumour CD8^+^ T cells from an exhausted effector-like state to a more memory-like state. These results reveal a mechanism of action through which TIGIT checkpoint inhibitors can remodel immunosuppressive tumour microenvironments, and suggest that FcγR engagement is an important consideration in anti-TIGIT antibody development.

## Abstract (web summary)

A high baseline of intratumoural macrophages and regulatory T cells is associated with better outcomes in patients with non-small cell lung cancer treated with atezolizumab plus tiragolumab, but not with atezolizumab alone.

## Main

PD-L1 blockade is efficacious in a broad range of malignancies. However, not all patients benefit, and a considerable fraction of initial responders eventually relapse^3–5^. One approach to extend and expand the impact of cancer immunotherapy has been to target additional immune checkpoints such as TIGIT (also known as T cell immunoreceptor with Ig and ITIM)^6^.

Atezolizumab is a PD-L1-targeting monoclonal antibody approved as first-line monotherapy for patients with metastatic non-small cell lung cancer (NSCLC) whose tumours have high PD-L1 expression, and as an adjuvant treatment in patients with resected stage II–IIIA NSCLC^7,8^. Tiragolumab is a monoclonal antibody that binds to TIGIT and prevents it from binding to the high-affinity ligand PVR (also known as CD155) as well as to its counter-receptor CD226^1,6^. In the randomized phase 2 study CITYSCAPE, we evaluated the efficacy of first-line tiragolumab plus atezolizumab versus atezolizumab monotherapy in patients with PD-L1-positive (tumour proportion score (TPS) ≥ 1%) NSCLC. The combination treatment demonstrated superior clinical benefit, with an objective response rate (ORR) of 31% versus 16% in individuals treated with atezolizumab plus placebo, and an improvement in progression-free survival (PFS) (hazard ratio (HR) = 0.62, 95% confidence interval (CI) = 0.42–0.91) and overall survival (OS) (HR = 0.69, 95% CI = 0.44–1.07) in the intent-to-treat population^1^.

In mouse models, the TIGIT and PD-1 pathways are mechanistically interdependent, and co-blockade of TIGIT and PD-L1 has been shown to synergistically elicit anti-tumour T cell responses^9,10^. Several mechanisms of action have been proposed for TIGIT targeted therapies, including Fc-independent receptor–ligand blockade, Fc-dependent depletion of TIGIT-expressing regulatory T (T_reg_) cells and Fc-dependent myeloid cell modulation^9–16^. It is unclear which of these mechanisms are relevant in the clinical blockade of TIGIT, and the functionality of the anti-TIGIT Fc domain has been the subject of debate^2^.

Here, in a clinical biomarker analysis of anti-TIGIT and anti-PD-(L)1 antibody combination immunotherapy, coupled with preclinical exploration, we identify a mechanism of action for tiragolumab and suggest that Fc domain functionality is important in anti-TIGIT antibodies.

## Tiragolumab benefits from TAMs and T_reg_ cells

**Fig. 1:**
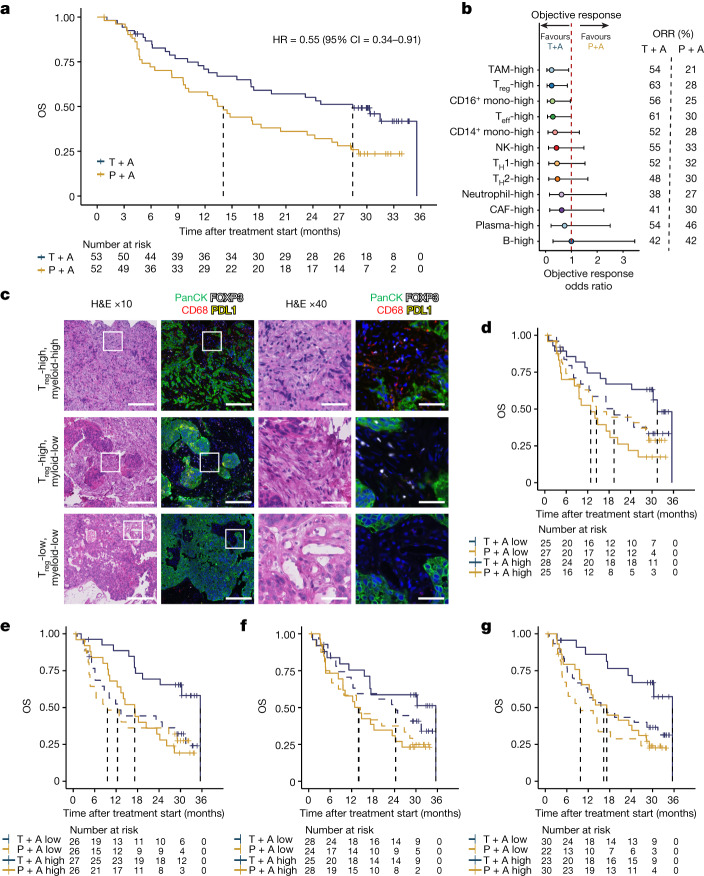
Intratumoural myeloid and T_reg_ cell content is associated with patient benefit after combination treatment with tiragolumab plus atezolizumab in the CITYSCAPE trial. **a**, Kaplan–Meier curve comparing the OS of patients in the BEP who received tiragolumab + atezolizumab (blue) or placebo + atezolizumab (gold). **b**, Comparison of the overall objective response odds ratio of tiragolumab + atezolizumab versus placebo + atezolizumab in patients whose tumours had high cell type abundance. Intratumoural cell types were determined as high or low on the basis of the median signature score cut-offs. Odds ratio calculations were performed using Fisher’s exact tests. The dots represent the objective response odds ratio and the horizontal bars show the 95% CI. **c**, Multiplex immunofluorescence staining of pan-cytokeratin (panCK; green), FOXP3 (white), CD68 (red), and PD-L1 (yellow) in CITYSCAPE patient tumour samples (*n* = 27). Representative images are shown for T_reg_-high/myeloid-high (top), T_reg_-high/myeloid-low (middle) and T_reg_-low/myeloid-low (bottom). Scale bars, 200 μm (columns 1 and 2) and 50 μm (columns 3 and 4). H&E, haematoxylin and eosin. **d**–**g**, Kaplan–Meier curves comparing the OS in patients with tumours enriched (solid lines) or not enriched (dashed lines) for the top four cell types in **b**, including TAMs (**d**), T_reg_ cells (**e**), CD16^+^ monocytes (**f**) and CD8^+^ T effector (T_eff_) cells (**g**), that were associated with response to tiragolumab + atezolizumab. Enrichment or not was determined by the median cell type signature score cut-offs. For **a** and **d**–**g**, HRs and 95% CIs were determined using a univariate Cox model. CAFs, cancer-associated fibroblasts; mono, monocytes; P + A, placebo + atezolizumab; T + A, tiragolumab + atezolizumab; T_H_, T helper cells.

**Extended Data Fig. 1:**
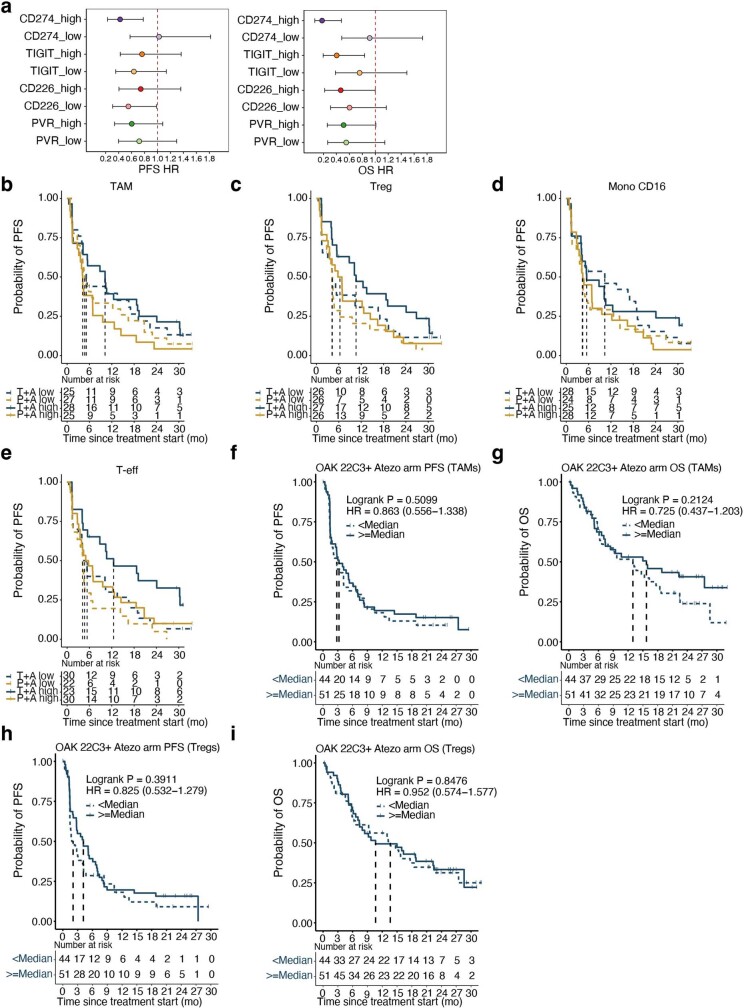
Intratumoural myeloid and Treg cell content correlates with tiragolumab plus atezolizumab outcome but not placebo plus atezolizumab. **a**, Forest plot comparing tiragolumab plus atezolizumab versus placebo plus atezolizumab in patients with tumours expressing high or low gene levels (cutoff by median expression) of *CD274*, *TIGIT*, *CD226*, and *PVR* in CITYSCAPE. Hazard ratio and 95% confidence interval were determined using univariate Cox model. The dots represent the hazard ratio and the horizontal bars the 95% confidence interval. **b–e**, Kaplan–Meier curves comparing PFS in patients with tumours enriched (solid lines) or not enriched (dashed lines) for TAMs (**b**), Tregs (**c**), CD16-high monocytes (**d**), and CD8 + T effector cells (T-eff) (**e**). Enrichment or not was determined by the median cell type signature score cutoffs. **f, g**, Kaplan–Meier curves comparing the PFS (**f**) and OS (**g**) in PD-L1-positive patients from the phase 3 NSCLC OAK study who received atezolizumab monotherapy and had tumours enriched for TAMs. **h, i**, Kaplan–Meier curves comparing the PFS (**h**) and OS (**i**) in PD-L1-positive patients from the phase 3 NSCLC OAK study who received atezolizumab monotherapy and had tumours enriched for Tregs. **f-i**, Hazard ratio and 95% confidence interval were determined using univariate Cox model, and *P* values were estimated using the log-rank test.

We performed bulk RNA sequencing (RNA-seq) analysis of pretreatment tumour samples from patients enrolled in the CITYSCAPE trial. This biomarker-evaluable population (BEP, *n* = 105) displayed comparable baseline demographics to the intent-to-treat population (*n* = 135; Supplementary Table 1), and similar benefits of tiragolumab plus atezolizumab therapy with a BEP OS HR of 0.55 (95% CI = 0.34–0.91; Fig. 1a)^1^. Consistent with the results of our post hoc analysis of PD-L1 immunohistochemistry^1^, high *CD274* gene expression was associated with improved PFS and OS in the tiragolumab plus atezolizumab arm compared with the placebo plus atezolizumab arm (PFS HR = 0.42, 95% CI = 0.23–0.78; OS HR = 0.18, 95% CI = 0.06–0.48; Extended Data Fig. 1a).

**Extended Data Fig. 2:**
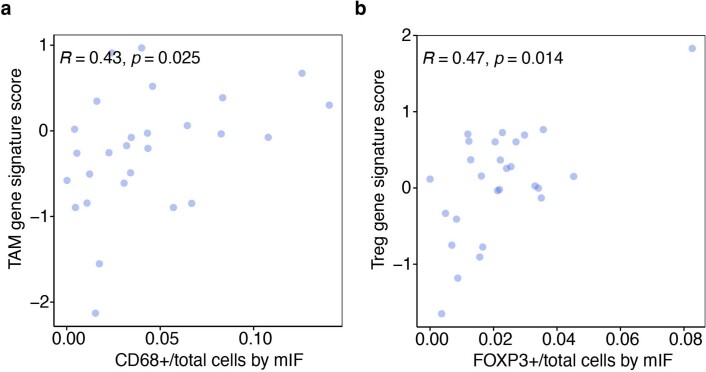
Correlation of bulk RNA-seq-based cell type signature scores with multiplex immunofluorescence. **a, b**, Correlation of TAM signature with CD68+ cells by mIF (**a**) and Treg signature with FoxP3+ cells by mIF (**b**). Two-tailed Pearson correlation; n = 27. mIF, multiplex immunofluorescence.

To investigate the mechanisms underlying the tiragolumab plus atezolizumab combination benefit, we stratified patients on the basis of intratumoural leukocyte and stromal cell gene expression signatures and evaluated the association of each signature with clinical outcome. Consistent with their central role in the efficacy of other checkpoint inhibitors, CD8^+^ effector T cells were associated with an improved ORR in patients treated with tiragolumab plus atezolizumab (Fig. 1b). Unexpectedly, a higher abundance of tumour-associated macrophages (TAMs) and T_reg_ cells, which function as immunosuppressive cells in the tumour microenvironment, was also associated with improved ORR in the combination regimen relative to the control arm (Fig. 1b and Supplementary Table 2). To confirm our TAM and T_reg_ cell transcriptional findings, we evaluated pretreatment tumour samples (*n* = 27) using multiplex immunofluorescence staining for pan-cytokeratin (tumour marker), FOXP3 (T_reg_ cell marker), CD68 (macrophage marker) and PD-L1. Abundant CD68^+^ cells and FOXP3^+^ cells were detected in samples with high TAM and T_reg_ cell signatures measured by bulk RNA-seq (Fig. 1c), which also exhibited positive correlations with cell counts measured by multiplex immunofluorescence staining (Extended Data Fig. 2a,b).

Kaplan–Meier analysis showed that increased TAMs and T_reg_ cells in the tumour were associated with improved OS for the combination treatment, but not for atezolizumab monotherapy: OS HR = 0.35 (95% CI = 0.17–0.73) for TAMs and OS HR = 0.31 (95% CI = 0.14–0.67) for T_reg_ cells (Fig. 1d,e). Monocytes, particularly CD16^high^ non-classical monocytes, also exhibited a positive association with survival in the tiragolumab plus atezolizumab treatment group (Fig. 1b,f). Increased CD8^+^ effector T cells were positively associated with treatment benefit in both arms (Fig. 1g). Combination benefit was not associated with B cells and plasma cells, which we and others have previously reported are strongly associated with atezolizumab clinical benefit^17^ (Fig. 1b). Similar associations were observed for PFS (Extended Data Fig. 1b–e).

We also analysed TAM and T_reg_ cell signatures in a similar patient population (PD-L1-positive (TPS ≥ 1%) NSCLC) in a larger independent dataset from the phase 3 OAK study^18^. Consistent with the atezolizumab plus placebo results in CITYSCAPE, TAM and T_reg_ cell signatures were not associated with improved PFS or OS with atezolizumab monotherapy in OAK (Extended Data Fig. 1f–i). Together, these data indicate that the treatment efficacy of tiragolumab plus atezolizumab combination was selectively albeit counterintuitively associated with TAMs and tumour T_reg_ cells, in addition to typical correlates of checkpoint inhibitor responsiveness such as CD8^+^ effector T cells and PD-L1 expression. We hypothesized that tiragolumab functioned as both a canonical checkpoint inhibitor as well as through an additional mechanism of action.

## Serum myeloid proteins linked to tiragolumab benefit

**Fig. 2:**
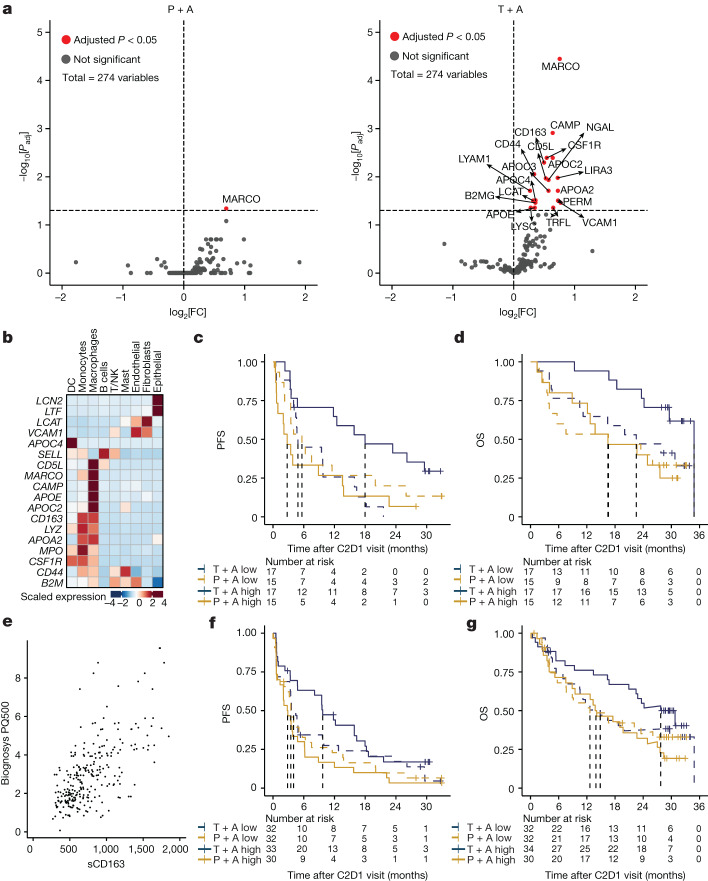
Treatment with tiragolumab plus atezolizumab leads to increased serum myeloid proteins. **a**, Differential abundance analysis of serum proteins at C2D1 relative to the baseline in patients who were treated with placebo + atezolizumab (left) or tiragolumab + atezolizumab (right). Statistical analysis was performed using limma with Benjamini–Hochberg correction. FC, fold change; *P*_adj_, adjusted *P*. **b**, The gene expression profiles of the significantly increased proteins in **a** based on a public NSCLC scRNA-seq dataset, suggesting a myeloid cell origin for most of these proteins, including NGAL (*LCN2*), TRFL (*LTF*), LCAT, VCAM1, APOC4, LYAM1 (*SELL*), CD5L, MARCO, CAMP, APOE, APOC2, CD163, LYSC (*LYZ*), APOA2, PERM (*MPO*), CSF-1R, CD44, B2MG (*B2M*); for protein–gene pairs that have distinct names, the gene names are shown in parentheses in italics. **c**,**d**, Kaplan–Meier curves of PFS (**c**) and OS (**d**) in patients with low (dashed lines) or high (solid lines) levels of serum myeloid proteins at C2D1 relative to C1D1 using a composite of significantly increased myeloid proteins (MARCO, CAMP, CD163, CSF-1R, CD5L, NGAL (*LCN2*), GAPR1, APOC1, APOC2, APOC3 and APOC4), as determined by the median composite score cut-off. **e**, The correlation between sCD163 levels by ELISA and CD163 detected by mass spectrometry. *n* = 266. Statistical analysis was performed using two-tailed Pearson correlation; *r* = 0.657, *P* < 2.2 × 10^−16^. **f**,**g**, Kaplan–Meier curves of the PFS (**f**) and OS (**g**) in patients with a low (dashed lines) and high (solid lines) fold change in sCD163 at C2D1 relative to C1D1, as determined by the median fold-change cut-off. For **c**,**d**,**f**,**g**, HRs and 95% CIs were determined using a univariate Cox model. DCs, dendritic cells. Source Data

As on-treatment tumour biopsies were not available, we used longitudinally collected peripheral serum samples to identify on-treatment signals associated with combination treatment in the CITYSCAPE trial. Mass spectrometry was used to profile serum proteins present on cycle 1 day 1 (C1D1, baseline) and cycle 2 day 1 (C2D1, 3 weeks after treatment) from serum samples of CITYSCAPE patients (*n* = 64). A comparison of circulating peptides at C2D1 versus the baseline showed a statistically significant increase (adjusted *P* < 0.05) in peptides derived from myeloid-cell-expressed proteins such as macrophage receptor with collagenous structure (MARCO), CSF-1R, CD163, CAMP, CD5L and apolipoproteins APOC2/3/4 in the patients treated with tiragolumab plus atezolizumab but not in the patients treated with placebo plus atezolizumab (Fig. 2a). The myeloid-specific expression patterns of genes encoding those upregulated proteins were confirmed using public NSCLC single-cell RNA-seq (scRNA-seq) gene expression data (Fig. 2b).

To understand the kinetics of these proteins in the context of clinical outcomes, we generated a composite signature of significantly modulated myeloid proteins (*n* = 11) using their C2D1 fold changes relative to the baseline, and performed Kaplan–Meier survival analysis for PFS and OS (Fig. 2c,d). In patients who had a greater increase in these serum myeloid proteins at 3 weeks, we observed that the patients treated with the tiragolumab plus atezolizumab combination showed a longer PFS and OS than patients who received placebo plus atezolizumab (PFS HR = 0.32, 95% CI = 0.14–0.72; OS HR = 0.30, 95% CI = 0.11–0.81), suggesting that myeloid cell activation may be an important mechanism of response that is specific to the combination treatment.

Circulating serum soluble CD163 (sCD163) is a known marker of monocyte and tissue macrophage activation and is a haemoglobin–haptoglobin scavenger receptor expressed exclusively on monocytes and macrophages^19,20^. sCD163 was measured in available serum samples from CITYSCAPE patients (*n* = 132) using an sCD163 enzyme-linked immunosorbent assay (ELISA). sCD163 levels, as determined using an ELISA, were correlated with CD163 detected by mass spectrometry in patients for whom there were both sets of data (Fig. 2e). Using the C2D1 fold changes relative to the baseline, Kaplan–Meier survival analysis for PFS and OS showed that, in patients with greater elevation in sCD163, tiragolumab plus atezolizumab combination treatment conferred an improved PFS and OS compared with treatment with placebo plus atezolizumab (PFS HR = 0.47, 95% CI = 0.29–0.80; OS HR = 0.49, 95% CI = 0.29–0.91; Fig. 2f,g).

## Tiragolumab activates peripheral monocytes

**Fig. 3:**
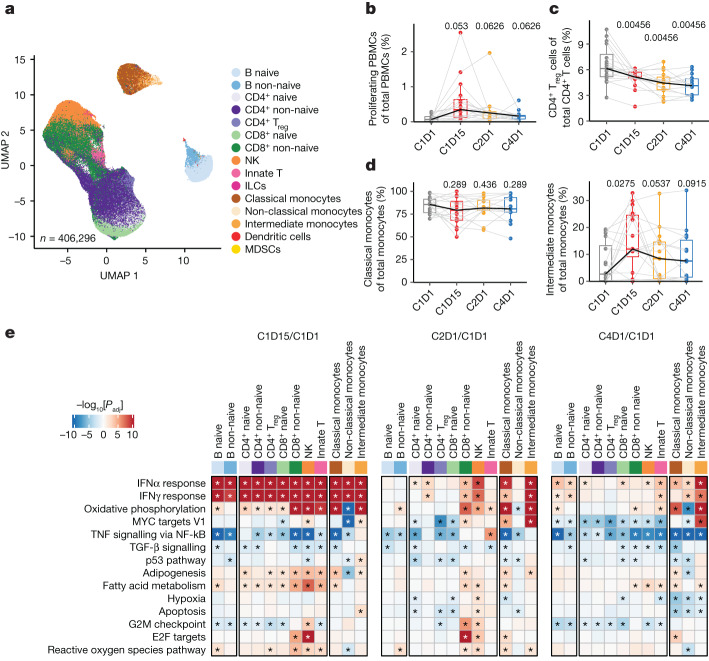
Tiragolumab plus atezolizumab leads to T, NK and myeloid cell activation in PBMCs. **a**, Uniform manifold approximation and projection (UMAP) analysis of PBMC single cells coloured by cell types. *n* = 406,296. **b**, The proportion of proliferating cells in PBMCs over different timepoints. *n* = 16. **c**, The proportion of T_reg_ cells out of total CD4^+^ T cells over different timepoints. *n* = 16. **d**, The proportion of classical monocytes (left) and intermediate monocytes (right) out of total monocytes over different timepoints. *n* = 16. **e**, Pathway enrichment in PBMC samples obtained on-treatment compared with those obtained at the baseline across multiple immune cell types from patients with NSCLC. *n* = 16. Enrichment in on-treatment (red) and baseline (blue) samples is indicated. *P* values were calculated using nonparametric permutation tests; the asterisks represent false-discovery rate < 0.05. For the box plots in **b**–**d**, the centre line shows the median, the box limits show the interquartile range (IQR; the range between the 25th and 75th percentile) and the whiskers show 1.58 × IQR. Median values per timepoint are connected by solid black lines; samples from the same patient are connected by grey lines. *P* values were calculated using two-tailed paired Student’s *t*-tests and adjusted using the Benjamini–Hochberg procedure. ILCs, innate lymphoid cells; MDSCs, myeloid-derived suppressor cells. Source Data

**Extended Data Fig. 3:**
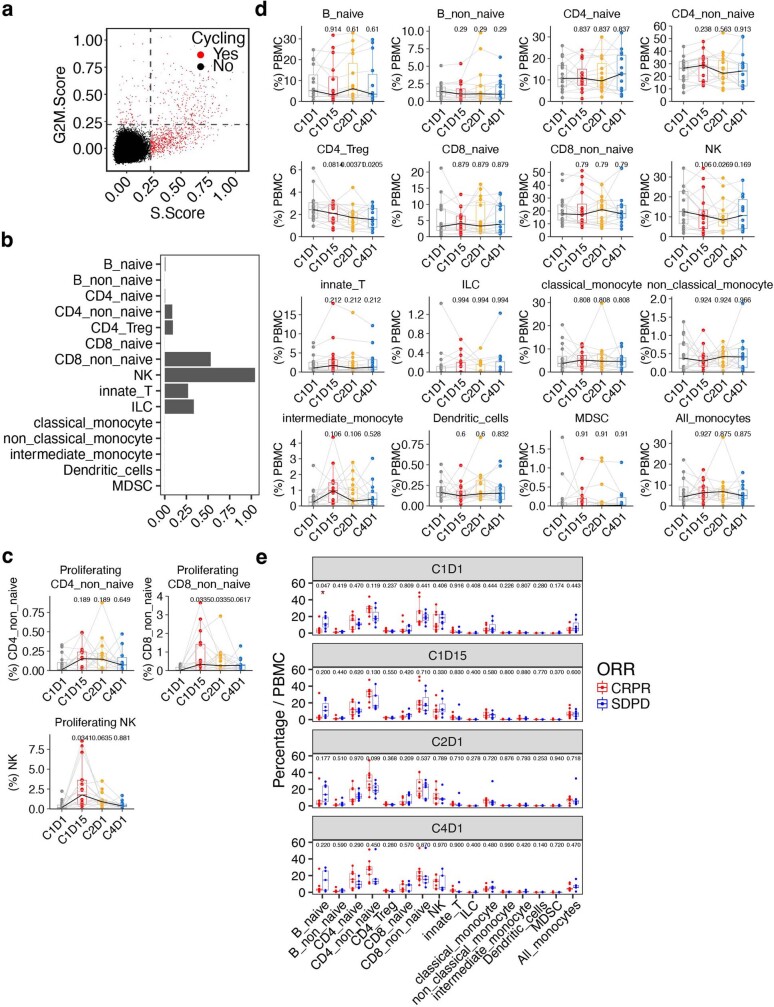
The proportion of proliferation cells and major cell types in PBMC. **a**, Scatter plot showing the S and G2M cell cycle phase scores, coloured by cells in proliferating (red) or non-proliferating states (black). **b**, Bar plot showing the proportion of proliferating cells in each major cell type. **c**, Box plots showing the proportion of proliferating cells in CD4_non_naive, CD8_non_naive, and NK cells, across each timepoint. **d**, Box plots comparing the proportions of each cell type at on-treatment (C1D15, C2D1, and C4D1) versus baseline (C1D1). **e**, Box plots comparing the proportions of each cell type between responders and non-responders at baseline (C1D1) and on-treatment (C1D15, C2D1, and C4D1). **c-e**, Boxplot center line, median; box, interquartile range (IQR; the range between the 25th and 75th percentile); whiskers, 1.58 × IQR. **c,d**, Median values per time point are connected by solid black lines; samples from the same patient at different time points are connected by grey lines. **c-d**, *P* values shown were calculated by two-tailed paired Student’s *t*-test and BH-adjusted. **e**, Nominal *P* values derived from two-tailed unpaired Student *t*-test are shown and red asterisk represents significance levels where * *P* < 0.05. **c-e**, n = 16 patients. Source Data

We evaluated the effects of tiragolumab plus atezolizumab therapy on peripheral blood mononuclear cells (PBMCs) collected at C1D1, C1D15 (2 weeks after the initial treatment), C2D1 (3 weeks after the initial treatment) and C4D1 (9 weeks after the initial treatment) from patients in the phase 1b NSCLC study of tiragolumab plus atezolizumab (GO30103)^21^. Using scRNA-seq and CITE-seq, transcriptional profiles of 406,296 immune cells were obtained and annotated (Fig. 3a). We observed increased proliferation of peripheral cells at C1D15 (Fig. 3b and Extended Data Fig. 3a), especially in the subsets of non-naive CD8^+^ cells and natural killer (NK) cells (Extended Data Fig. 3b,c). The proportions of major cell types as a fraction of PBMCs were not altered during the treatment (Extended Data Fig. 3d) or between responders and non-responders at each timepoint (Extended Data Fig. 3e). The proportion of circulating T_reg_ cells decreased under treatment when evaluated as the fraction of total CD4^+^ T cells (Fig. 3c). Notably, intermediate monocytes increased at C1D15 while classical monocytes appeared to decrease when evaluated as a fraction of total monocytes (Fig. 3d).

Gene set enrichment analysis comparing changes at C1D15 relative to the baseline (C1D1) using the Hallmark collection^22^ showed a broad interferon (IFN) response in all cell types; then, at C2D1, the response appeared to become more specific. Increased IFN signalling was observed in non-naive CD8^+^ and CD4^+^ T cells, NK cells and monocytes, consistent with previous observations for atezolizumab monotherapy^23,24^ (Fig. 3e). We also observed some novel pathways upregulated in monocytes, including the oxidative phosphorylation pathway and the MYC-targeting pathway, which has been shown to regulate macrophage polarization^25^. Taken together, these data suggest that myeloid cell activation is an important component of tiragolumab activity. Given tiragolumab’s active Fc and high levels of FcγR expression by myeloid cells, we further hypothesized that FcγR engagement could contribute to the antibody’s anti-tumour efficacy.

## Anti-TIGIT remodels the TME through FcγR

We turned to preclinical models to study the effects of anti-TIGIT and Fc–FcγR interactions on tumour-infiltrating leukocytes. For mouse modelling, we selected the syngeneic tumour model CT26, which has been used in previous studies of TIGIT antibody function and is infiltrated by T cells and myeloid cells at levels that are comparable to those in human NSCLC^11–13^. Tumour-bearing mice were treated with mouse-reactive surrogate anti-TIGIT monoclonal antibodies bearing varying Fc domains: mIgG2a-LALAPG (Fc inert), which lacks effector function^26^, mIgG2b, which engages activating and inhibitory FcγR, and mIgG2a, which preferentially engages activating FcγR^27^.

**Fig. 4:**
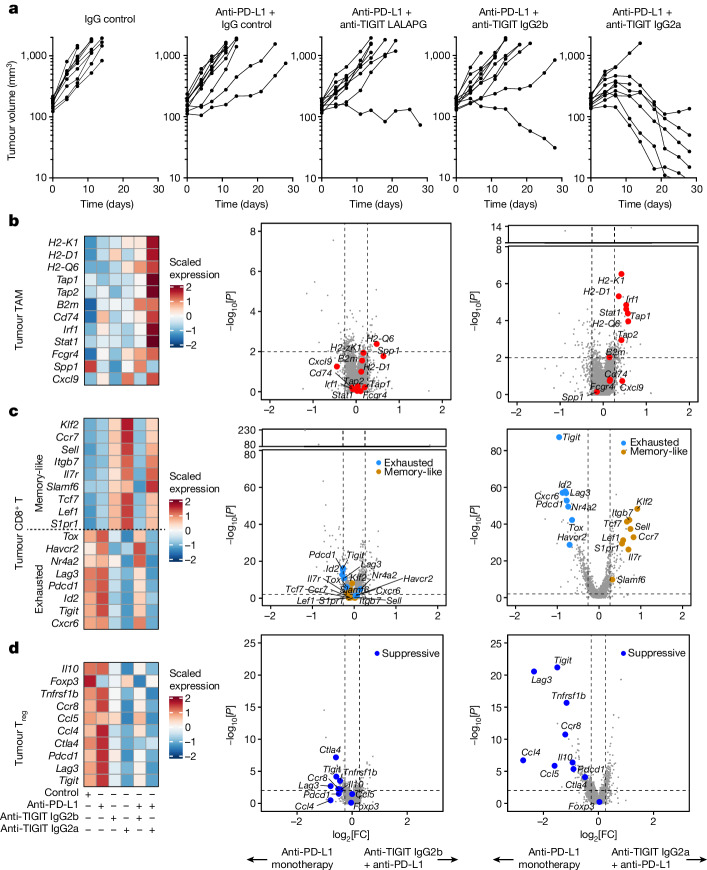
Fc receptor engagement supports tiragolumab surrogate efficacy and ability to remodel the tumour microenvironment in mice. **a**, Growth of CT26 tumours in syngeneic BALB/c mice under various treatments. Data are representative of two or more independent experiments with *n* = 10 mice in each group. **b**–**d**, Heat maps of the expression of selected genes across different treatments in tumour macrophages and monocytes combined (**b**; left), tumour CD8^+^ T cells combined (**c**; left) and tumour CD4^+^ T_reg_ cells (**d**; left). Volcano plots showing gene expression for anti-PD-L1 + anti-TIGIT IgG2b versus anti-PD-L1 (middle), and anti-PD-L1 + anti-TIGIT IgG2a versus anti-PD-L1 (right) in tumour macrophages and monocytes combined (**b**), tumour CD8^+^ T cells combined (**c**) and tumour CD4^+^ T_reg_ cells (**d**). For the volcano plots in **b**–**d**, the broken *y* axis was used to make the *y*-axis range comparable and for better comparison between treatments. *P* values were calculated using two-tailed Wilcoxon rank-sum tests. Source Data

**Extended Data Fig. 4:**
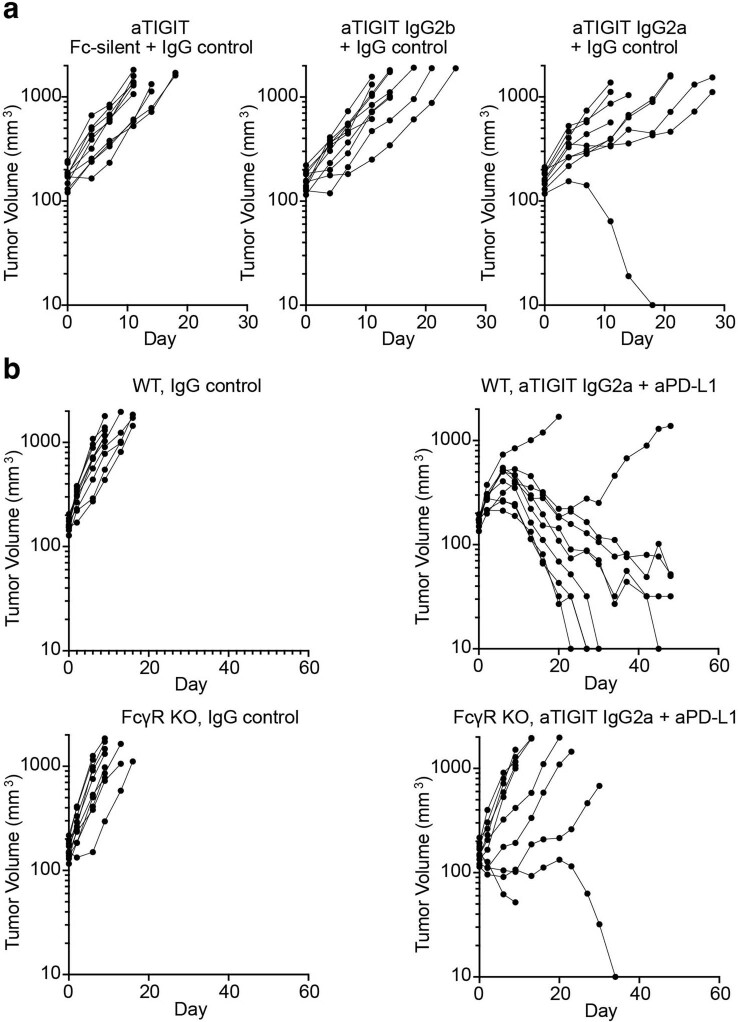
Efficient tumour rejection by anti-PD-L1 and anti-TIGIT mAbs treatment depends on functional Fc-FcɣR interaction axis. **a**, Plots depicting tumour volumes in each mouse over time; data are representative of one independent experiment. Wildtype BALB/c mice were implanted with CT26 tumours and then treated as described in the method. **b**, Plots depicting tumour volumes in each mouse over time; data are representative of one independent experiment. Wildtype (top) and FcɣR knockout (bottom) BALB/c mice were implanted with CT26 tumours and then treated as described in the method. Source Data

We first investigated the efficacy of anti-TIGIT Fc variants in controlling tumour growth. mIgG2a anti-TIGIT, but not mIgG2b or Fc-inert anti-TIGIT, antibodies were capable of inducing tumour rejection when combined with Fc inert mouse anti-PD-L1 (Fig. 4a). Anti-TIGIT monotherapies, including the mIgG2a-formatted monoclonal antibody, exhibited a limited effect on controlling tumour growth (Extended Data Fig. 4a). The combination of mIgG2a anti-TIGIT and anti-PD-L1 antibodies did not control tumour growth in FcγR-knockout mice, confirming a requirement for Fc–FcγR engagement for therapeutic activity of anti-TIGIT monoclonal antibodies in this model (Extended Data Fig. 4b).

**Extended Data Fig. 5:**
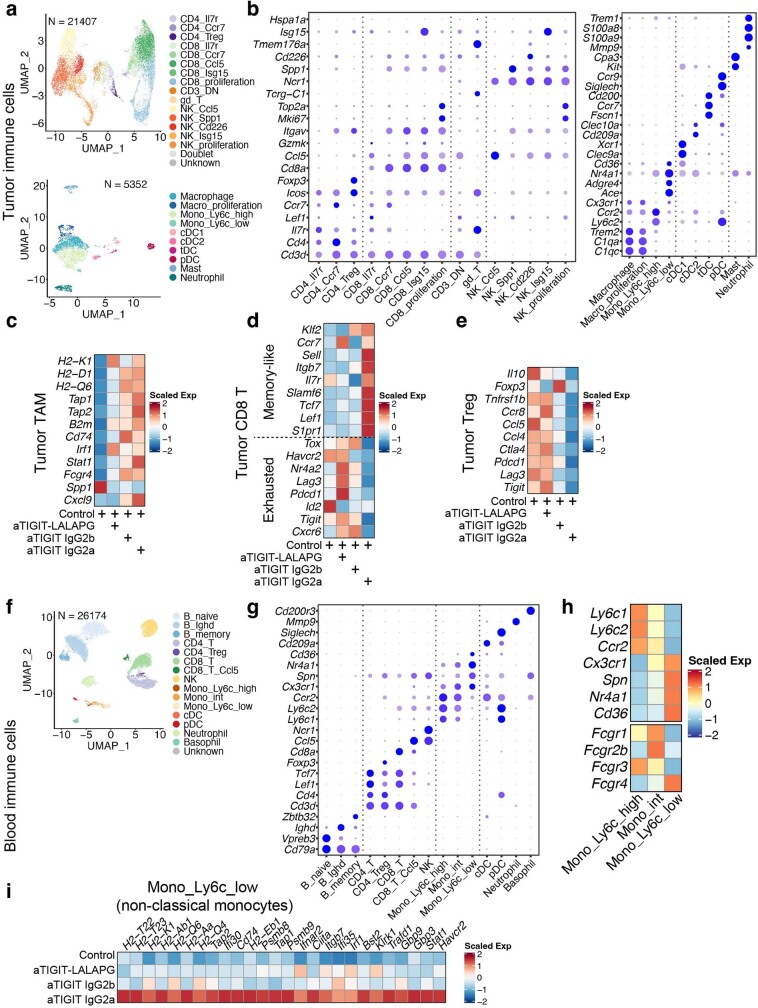
anti-TIGIT treatment modulation of tumour infiltrating immune cells and peripheral blood monocytes depends on the Fc region. **a**, UMAP of single cells from tumour infiltrating T and NK cells (top, n = 21,407) and myeloid cells (bottom, n = 5,352) coloured by cell types. **b**, Bubble plots showing marker gene expression for T and NK cells (left) and myeloid cells (right) as shown in (**a**).**c-e**, Heatmaps showing the expression of selected genes across different treatments in tumour macrophages and monocytes combined (**c**), tumour CD8 + T cells combined (**d**), and tumour CD4+ Tregs (**e**). **f**, UMAP of single cells from the peripheral blood (n = 26,174) coloured by cell types. **g**, Bubble plots showing the marker gene expression of cell types as in (**f**). **h**, Heatmap displaying the scaled gene expression of marker genes distinguishing classical, non-classical, and intermediate monocytes, and the expression patterns of FcɣR. **i**, Heatmap showing the scaled gene expression of MHC and interferon response in non-classical monocytes across different treatments. **a-i**, Single cell RNA-seq was performed on intratumoural (**a-e**) and peripheral (**f-i**) CD45+ cells isolated at day 3 after treatment, and data are from one independent experiment with n = 5 mice in each group. Source Data

Next, we assessed the effects of anti-TIGIT Fc variants on tumour-infiltrating and blood leukocytes using scRNA-seq. From within the tumours, we characterized 21,407 T and NK cells, and 5,352 myeloid cells (Extended Data Fig. 5a,b). Gene expression analysis of tumour macrophages and monocytes revealed that IgG2a anti-TIGIT and IgG2b anti-TIGIT antibodies both increased the expression of antigen-presentation genes, with IgG2a anti-TIGIT antibodies having a greater effect (Extended Data Fig. 5c). Analysing tumour CD8^+^ T cells, we found that treatment with Fc-inert anti-TIGIT antibodies increased the expression of genes associated with effector differentiation and exhaustion such as *Pdcd1*, *Lag3* and *Tox*, while expression of those same genes was reduced by treatment with Fc-active IgG2a anti-TIGIT antibodies (Extended Data Fig. 5d). Concurrently, IgG2a anti-TIGIT antibodies increased the expression of genes associated with a memory-like or T stem-cell-like state such as *Tcf7* (Extended Data Fig. 5d). Within tumour CD4^+^ T_reg_ cells, IgG2b and IgG2a anti-TIGIT antibodies reduced the expression of genes associated with suppressive capacity, including *Tigit*, *Ccr8* and *Ctla4* (Extended Data Fig. 5e). We also characterized 26,174 circulating leukocytes, with a particular interest in the non-classical monocytes found to be associated with tiragolumab + atezolizumab outcomes in CITYSCAPE (Extended Data Fig. 5f–h). We found that IgG2a anti-TIGIT, but not Fc-inert or IgG2b anti-TIGIT, drove increased expression of antigen-presentation and IFN-response genes in non-classical monocytes (Extended Data Fig. 5i).

**Extended Data Fig. 6:**
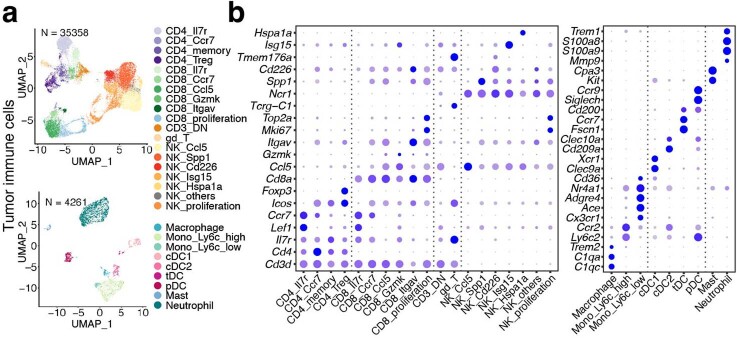
Annotation of single cells collected from mouse tumours. Single cell RNA-seq was performed on intratumoural CD45+ cells isolated from tumours at day 3 after treatment, and data are from one independent experiment with n = 5 mice in each group. This is related to Fig. 4b–d. **a**, UMAP of tumour-infiltrating lymphocytes (top, n = 35,358) and myeloid (bottom, n = 4,261) cells coloured by cell types. **b**, Bubble plots showing marker gene expression for T and NK cells (left) and myeloid cells (right) as shown in **(a)**. Source Data

We next investigated the pharmacodynamic effects of Fc-active anti-TIGIT antibody treatment in combination with anti-PD-L1 antibodies. Tumour-bearing mice were treated with control, Fc-inert anti-PD-L1, and/or IgG2a and IgG2b anti-TIGIT antibodies, and tumour-infiltrating leukocytes were captured and sequenced using scRNA-seq (*n* = 35,358 (tumour T and NK cells) and *n* = 4,261 (tumour myeloid cells); Extended Data Fig. 6a,b). Although anti-PD-L1 monotherapy had little effect, anti-PD-L1 + IgG2a anti-TIGIT combination treatment inflamed tumour macrophages, amplifying the antigen-presentation gene program induced by anti-TIGIT antibodies alone (Fig. 4b). Anti-PD-L1 + IgG2b anti-TIGIT did not elicit a comparable effect, consistent with the weaker effect of IgG2b anti-TIGIT antibodies relative to the IgG2a Fc variant (Fig. 4b). In tumour CD8^+^ T cells, treatment with anti-PD-L1 sustained expression of the gene program associated with exhaustion, characterized by the transcriptional regulators *Tox*, *Nr4a2* and *Id2* as well as the co-inhibitory receptors *Pdcd1*, *Tigit*, *Lag3* and *Havcr2*. By contrast, treatment with anti-TIGIT antibodies drove a shift in tumour CD8^+^ T cells away from that program and towards one associated with memory state, with elevated expression of *Tcf7*, *Klf2*, *Ccr7*, *Lef1*, *Il7r* and *Sell* (Fig. 4c). Treatment with IgG2a anti-TIGIT antibodies continued to drive this conversion towards memory-like cells, even in combination with anti-PD-L1 antibodies, while treatment with IgG2b anti-TIGIT plus anti-PD-L1 antibodies led to expression of the gene program observed with control or anti-PD-L1 monotherapy (Fig. 4c). In tumour T_reg_ cells, both anti-TIGIT isotypes drove downregulation of immunosuppressive and T_reg_-cell-associated genes such as *Il10*, *Ctla4* and *Tnfrsf1b* relative to treatment with anti-PD-L1 antibodies or control, and sustained those effects in combination with anti-PD-L1 (Fig. 4d).

**Extended Data Fig. 7:**
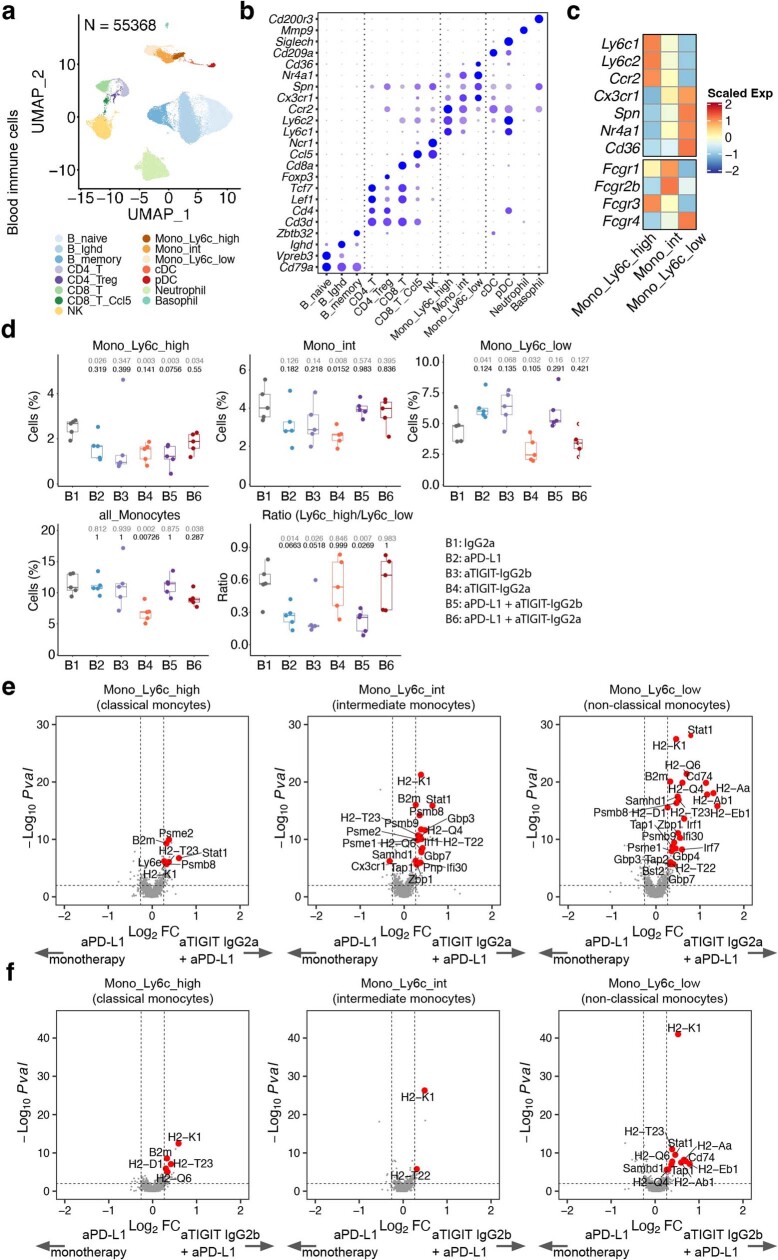
The modulation effects of anti-PD-L1 + anti-TIGIT on peripheral blood monocytes depends on the anti-TIGIT mAb Fc region. **a**, UMAP of single cells from the peripheral blood cells (n = 55,368) coloured by cell types. **b**, Bubble plot showing the marker gene expression of cell types as in (**a)**. **c**, Heatmap displaying the scaled gene expression of marker genes distinguishing classical, non-classical, and intermediate monocytes, and the expression patterns of FcɣR. **d**, Box plots comparing cell proportions of different treatments versus IgG2a isotype control (B1). B2, aPD-L1; B3, aTIGIT-IgG2b; B4, aTIGIT-IgG2a; B5, aPD-L1 + aTIGIT-IgG2b; B6, aPD-L1 + aTIGIT-IgG2a. Boxplot center line, median; box, interquartile range (IQR; the range between the 25th and 75th percentile); whiskers, 1.58 × IQR. Normal *P* values by two-tailed unpaired Student’s *t*-test are shown in grey colour; adjusted *P* values by Dunnett’s multiple comparison were shown in black colour. **e, f**, Volcano plots showing the gene expression of anti-PD-L1 + anti-TIGIT IgG2a versus anti-PD-L1 (**e**), and anti-PD-L1 + anti-TIGIT-IgG2b versus anti-PD-L1 (**f**) in peripheral blood classical (left), intermediate (middle), and non-classical (right) monocytes. *P* values were calculated by two-tailed Wilcoxon rank-sum test. **a-f**, Single cell RNA-seq was performed on peripheral CD45+ cells isolated at day 3 after treatment, and data are from one independent experiment with n = 5 mice in each group. Source Data

In the blood, a total of 55,368 cells were single-cell sequenced and annotated (Extended Data Fig. 7a,b). Treatment with IgG2a anti-TIGIT antibodies alone or in combination with anti-PD-L1 led to as much as a 50% decrease in the frequency of circulating monocytes relative to the control (Extended Data Fig. 7d). Non-classical monocytes appeared to be most affected, with decreased prevalence in mice treated with IgG2a anti-TIGIT antibodies but increased prevalence in mice treated with anti-PD-L1 and/or IgG2b anti-TIGIT antibodies (Extended Data Fig. 7c,d). Relative to treatment with anti-PD-L1 alone, combination treatment with IgG2a anti-TIGIT plus anti-PD-L1 antibodies led to a general induction of antigen-presentation programs in all monocyte subsets as well as more specific induction of IFN-response gene signatures in non-classical monocytes and intermediate monocytes (Extended Data Fig. 7e), which express higher levels of activating FcγR compared with classical monocytes (Extended Data Fig. 7c). Similar monocyte modulation was observed in the IgG2b anti-TIGIT plus anti-PD-L1 antibody combination, but with a much smaller effect size (Extended Data Fig. 7f).

**Fig. 5:**
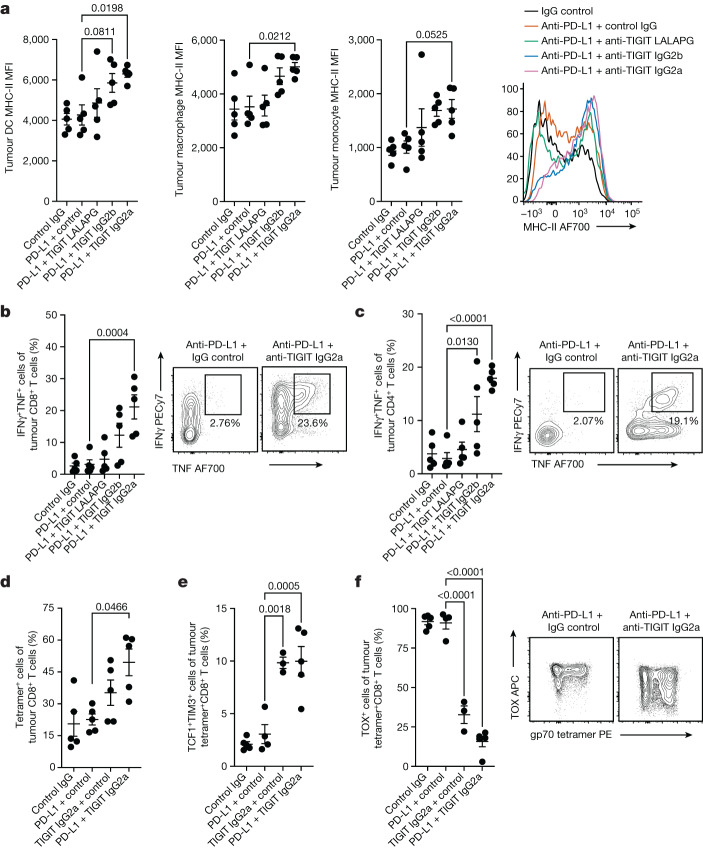
Flow cytometry analysis of anti-TIGIT antibody activity on tumour myeloid cells and lymphocytes. **a**, The mean fluorescence intensity (MFI) of cell surface MHC-II on tumour-infiltrating dendritic cells (left), macrophages (left middle) and monocytes (right middle). Right, histogram of representative surface MHC-II expression on tumour monocytes after various treatments. **b**, IFNγ and TNF co-expression in tumour-infiltrating CD8^+^ T cells after ex vivo stimulation (left). Right, representative fluorescence-activated cell sorting (FACS) analysis of tumour CD8^+^ T cell cytokine production after treatment with anti-PD-L1 monotherapy or anti-PD-L1 + anti-TIGIT IgG2a. **c**, IFNγ and TNF co-expression in tumour-infiltrating CD4^+^ T cells after ex vivo stimulation (left). Right, representative FACS analysis of CD4^+^ T cell cytokine production after treatment with anti-PD-L1 monotherapy or anti-PD-L1 + anti-TIGIT IgG2a. **d**, The frequency of gp70-tetramer-binding tumour CD8^+^ T cells. **e**, The frequencies of memory-like TCF1^+^TIM3^+^ gp70-tetramer-binding tumour CD8^+^ T cells. **f**, The frequencies of TOX^+^ gp70-tetramer-binding tumour CD8^+^ T cells (left). Right, representative FACS plots of tumour CD8^+^ T cell TOX expression and gp70 tetramer binding after treatment with anti-PD-L1 monotherapy or anti-PD-L1 + anti-TIGIT IgG2a. Intratumoural CD45^+^ cells were analysed using flow cytometry at day 3 after treatment (**a**–**c**) and gp70-tetramer-positive T cells at day 7 after treatment (**d**–**f**). Data are representative of one (**a**–**c**) or two (**d**–**f**) independent experiments with *n* = 5 mice in each group. For **a**–**f**, data are mean ± s.e.m. Statistical analysis was performed using one-way analysis of variance (ANOVA) with Dunnett’s multiple-comparison test, with the anti-PD-L1 monotherapy group designated as the control group. Source Data

**Extended Data Fig. 8:**
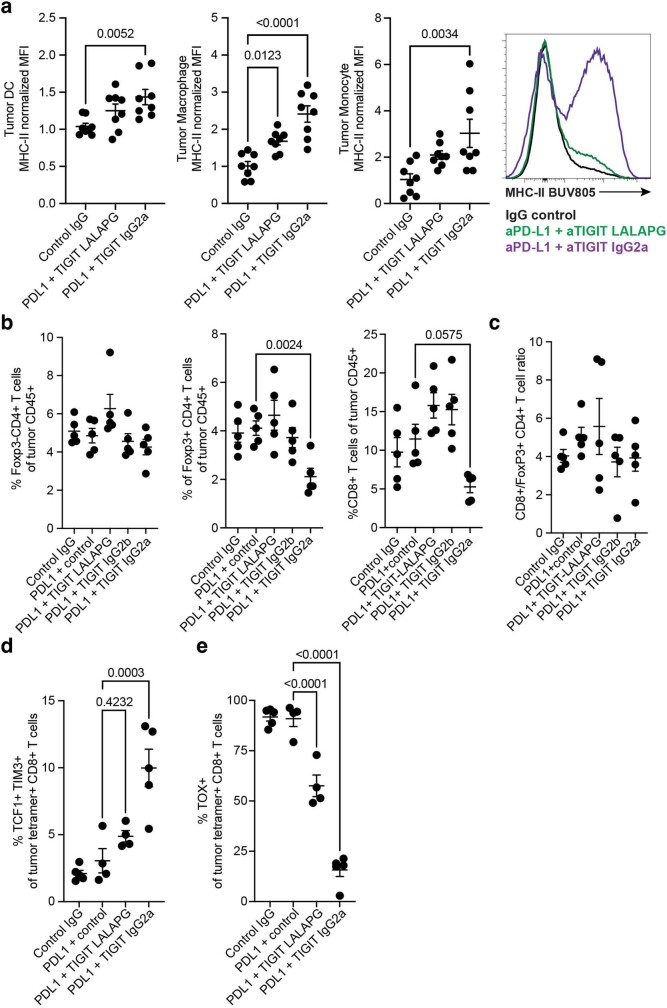
Flow cytometry analysis of anti-TIGIT activity in tumour myeloid cells of E0771 model, and T cells of CT26 model. **a**, Mean fluorescence intensity (MFI) of cell surface MHC-II on tumour-infiltrating dendritic cells (DC, left), macrophages (middle), and monocytes (right), normalized to their respective median MFI value following control treatment. Far right, histogram of representative surface MHC-II expression on tumour monocytes following various treatments. E0771-bearing C57BL/6 J mice were treated as indicated and data were collected at day 7 after treatment. Data are a composite of two independent experiments with n = 4 mice in each group; shown are mean +/− SEM with one-way ANOVA with Dunnett’s multiple comparisons, with the Control IgG group designated as the control group. **b**, Frequencies of tumour-infiltrating FoxP3- non-Treg CD4 + T cells (left), FoxP3+ Treg CD4 + T cells (middle), and CD8 + T cells (right) out of total CD45+ cells. **c**, Ratio of tumour CD8 + T cells to FoxP3+ Treg CD4 + T cells. **d, e**, Additional data related to Fig. 5e,f. Frequencies of TCF1 + TIM3+ memory-like T cells (**d**) and TOX+ terminally differentiated effector T cells (**e**) in CT26-tumour bearing mice treated with control and anti-PD-L1 plus anti-TIGIT mIgG2a-LALAPG or mIgG2a antibodies. **b-e**, Intratumoural CD45+ cells were analysed by flow cytometry at day 3 after treatment (**b, c**) and gp70 tetramer positive T cells at day 7 after treatment (**d, e**). Data are representative of one (**b, c**) or two (**d, e**) independent experiments with n = 5 mice in each group. **b-e**, Data in the dot plots are mean +/− SEM with one-way ANOVA with Dunnett’s multiple comparisons, with the anti-PD-L1 monotherapy group designated as the control group. Source Data

We found similar effects of Fc-active anti-TIGIT antibodies on the tumour microenvironment using flow cytometry. Consistent with the scRNA-seq analysis, Fc-active TIGIT monoclonal antibodies drove increased cell surface expression of MHC-II on tumour myeloid cells, including dendritic cells, macrophages and monocytes (Fig. 5a). We also observed this effect in the E0771 syngeneic model of triple-negative breast cancer (Extended Data Fig. 8a). FcγR engagement was also required for anti-TIGIT-mediated enhancement of CD8^+^ and CD4^+^ T cell competency to co-produce IFNγ and TNF, with IgG2a anti-TIGIT antibodies driving the strongest effects (Fig. 5b,c). IgG2a anti-TIGIT antibodies also induced moderate decreases in CD4^+^ T_reg_ cell and CD8^+^ T cell frequencies (Extended Data Fig. 8b), although the ratio of CD8^+^ T cells to T_reg_ cells was unchanged (Extended Data Fig. 8c). To assess the effects on tumour-antigen-specific immune responses, we analysed CD8^+^ T cells that recognized the CT26 tumour antigen gp70^28^. Treatment with anti-PD-L1 + mIgG2a anti-TIGIT antibodies increased the proportion of gp70-specific CD8^+^ T cells in the tumour (Fig. 5d) and drove those cells to downregulate TOX while upregulating TCF1, consistent with a shift from exhausted effector cells towards a more memory-like state (Fig. 5e,f). Fc-inert anti-TIGIT drove a lesser downregulation of TOX and upregulation of TCF1 (Extended Data Fig. 8d,e). Together, these data indicated that anti-TIGIT antibodies can engage activating Fc receptors to activate tumour macrophages, positively modulate tumour CD8^+^ and CD4^+^ T cells, and inflame circulating non-classical monocytes.

## Anti-TIGIT modulates CD8^+^ T cells via macrophages

Our data suggested macrophages and other myeloid cells might mediate the effects of anti-TIGIT antibodies on the anti-tumour T cell response. To test this hypothesis, we compared the effects of anti-PD-L1 + IgG2a anti-TIGIT treatment in the presence and absence of anti-CSF-1R, an antibody that functionally depletes macrophages and other myeloid cells that are reliant on CSF-1R signalling^29–31^. As macrophages are often seen as drivers of resistance to cancer immunotherapy, CSF-1R blockade and other macrophage-depleting therapeutic strategies are in clinical development and have been proposed as combination partners for checkpoint inhibitors^32^.

**Fig. 6:**
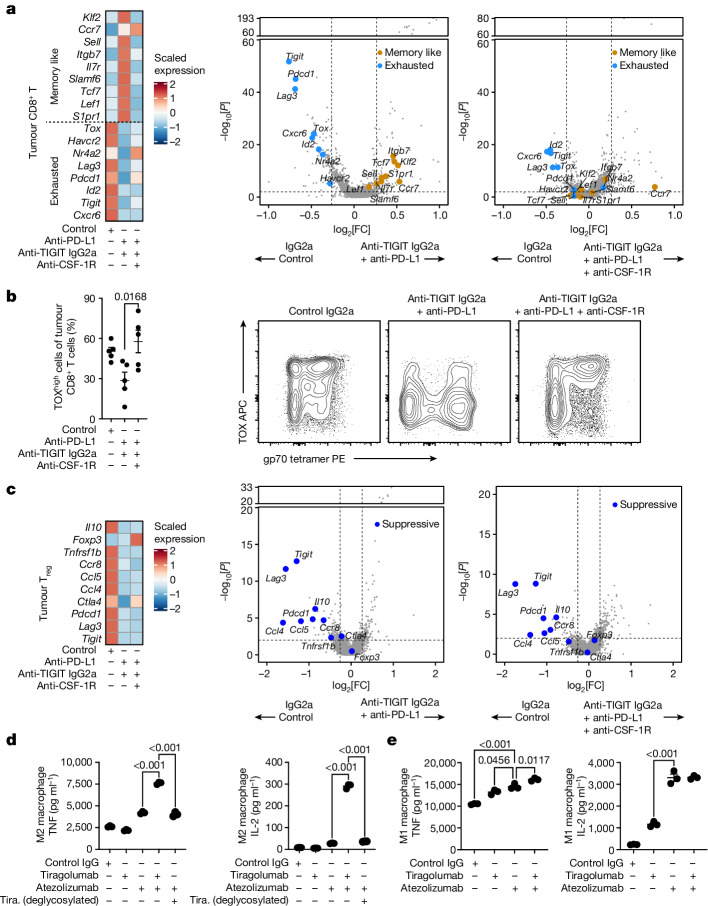
Macrophages enable modulation of CD8^+^ T cells by Fc-active anti-TIGIT antibodies in vivo and in vitro. **a**, Heat map showing the expression of selected genes across treatments in tumour CD8^+^ T cells (left). Volcano plots showing gene expression of tumour CD8^+^ T cells for anti-PD-L1 + anti-TIGIT IgG2a versus control IgG (middle) and anti-PD-L1 + anti-TIGIT IgG2a + anti-CSF-1R versus control IgG (right). **b**, The frequency of terminally differentiated TOX^high^ gp70-tetramer-binding tumour CD8^+^ T cells as measured using flow cytometry (left). Data are mean ± s.e.m. Statistical analysis was performed using one-way ANOVA with Dunnett’s multiple-comparison test, with the anti-PD-L1 + anti-TIGIT IgG2a group designated as the control group. Right, representative FACS plots from day 7 after treatment from two independent experiments (*n* = 5 mice) per group. **c**, The expression of selected genes across treatments in tumour CD4^+^ T_reg_ cells (left). Volcano plots showing gene expression of tumour CD4^+^ T_reg_ cells for anti-PD-L1 + anti-TIGIT IgG2a versus control IgG (middle) and anti-PD-L1 + anti-TIGIT IgG2a + anti-CSF-1R versus control IgG (right). For **a**,**c**, scRNA-seq analysis of intratumoural CD45^+^ cells at day 3 after treatment was from one independent experiment (*n* = 5 mice per group). In the volcano plots, the broken *y* axis was used to make the *y*-axis range comparable between treatments. *P* values were calculated using two-tailed Wilcoxon rank-sum tests (**a**,**c**). **d**,**e**, The effects of atezolizumab and tiragolumab (tira.) on the co-cultures of CMV-responsive PBMC T cells and M2-polarized (**d**) or M1-polarized (**e**) monocyte-derived macrophages as measured by TNF and IL-2 in the supernatant. Data are mean ± s.e.m., representative of two independent experiments with three PBMC donors per experiment. *P* values were calculated using one-way ANOVA with Tukey’s multiple-comparison test. Source Data

**Extended Data Fig. 9:**
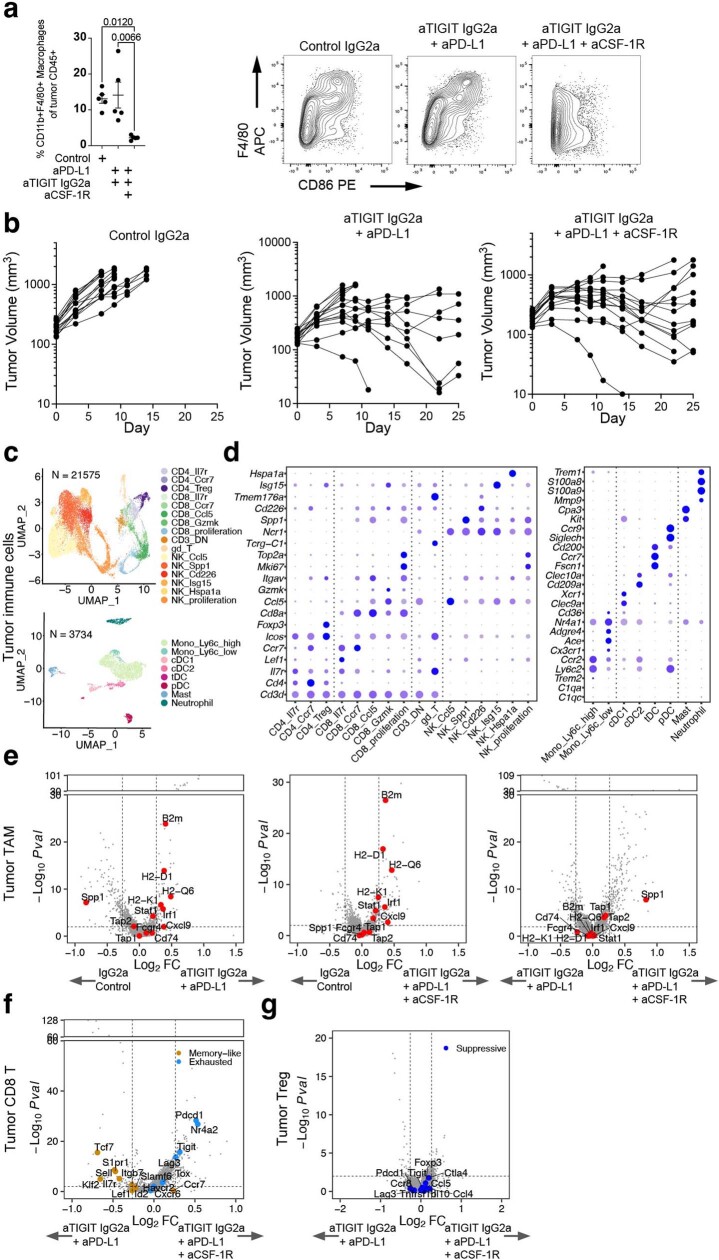
Tumour infiltrating leukocyte FACS and scRNA-seq analysis following treatment with anti-PD-L1, anti-TIGIT, and anti-CSF-1R. **a**, Percentage of tumour macrophages (left) and representative FACS plots of tumour CD11b+ cell expression of F4/80 and CD86 following treatment (right). Data were collected at day 7 after treatment, and are representative of two independent experiments with n = 5 mice in each group. Left, data are mean +/ − SEM with one-way ANOVA with Tukey’s multiple comparisons. **b**, Growth of CT26 tumours in syngeneic BALB/c mice treated with anti-gp120 (left), anti-PD-L1 + anti-TIGIT IgG2a (middle), and anti-PD-L1 + anti-TIGIT mIgG2a + anti-CSF-1R (right). Data are representative of two experiments with n = 10 mice in each group. **c**, UMAP of tumour-infiltrating lymphocytes (top, n = 21, 575) and myeloid (bottom, n = 3, 734) cells coloured by cell types. **d**, Bubble plots showing marker gene expression for T and NK cells (left) and myeloid cells (right) as shown in (**c**). **e**, Volcano plots showing the gene expression of anti-PD-L1 + anti-TIGIT IgG2a versus control IgG2a (left), anti-PD-L1 + anti-TIGIT IgG2a + anti-CSF-1R versus control IgG2a (middle), and anti-PD-L1 + anti-TIGIT IgG2a + anti-CSF-1R versus anti-PD-L1 + anti-TIGIT IgG2a (right) in tumour macrophage and monocytes combined. **f, g**, Volcano plots showing the gene expression of anti-PD-L1 + anti-TIGIT IgG2a + anti-CSF-1R versus anti-PD-L1 + anti-TIGIT IgG2a in tumour CD8 + T cells combined (**f**) and CD4 Tregs (**g**). **c-g**, Single cell RNA-seq was performed on intratumoural CD45+ cells isolated from tumours at day 3 after treatment, and data are from one independent experiment with n = 5 mice in each group. In volcano plots, the broken y-axis was used to make the y-axis range comparable and for better comparison between treatments; *P* values were calculated by two-tailed Wilcoxon rank-sum test. Source Data

In agreement with previous reports, the addition of anti-CSF-1R to anti-PD-L1 + anti-TIGIT partially depleted macrophages from the tumour beds of treated mice (Extended Data Fig. 9a) and sustained tumour responsiveness to checkpoint inhibitors (Extended Data Fig. 9b). Consistent with our previous experiments, scRNA-seq analysis of tumour CD8^+^ T cells showed that treatment with anti-PD-L1 + anti-TIGIT antibodies drove reduced expression of genes associated with exhaustion and increased expression of a memory-like gene program relative to single-agent treatment (Fig. 6a and Extended Data Fig. 9c,d). However, the addition of anti-CSF-1R treatment largely reversed this effect, reverting back towards the exhausted effector state (Fig. 6a and Extended Data Fig. 9e,f). We observed a similar result by flow cytometry, with anti-PD-L1 + anti-TIGIT treatment, but not anti-PD-L1 + anti-TIGIT + anti-CSF-1R treatment, driving a reduced expression of TOX in tumour CD8^+^ T cells (Fig. 6b). These data indicate that anti-TIGIT-mediated induction of a memory-like gene program in tumour CD8^+^ T cells is reliant on macrophages and other myeloid cells. By contrast, anti-CSF-1R treatment did not reduce the effects of anti-PD-L1 + anti-TIGIT treatment on tumour T_reg_ cells (Fig. 6c and Extended Data Fig. 9g), suggesting an alternative mechanism of action^6^.

To test the ability of tiragolumab to affect human CD8^+^ T cell responses through macrophages and FcγR engagement, we co-cultured PBMC-derived Tcells responsive to cytomegalovirus (CMV) with monocyte-derived macrophages, CMV peptides, atezolizumab and wild-type tiragolumab (that is, Fc-active) or deglycosylated tiragolumab (that is, Fc-silenced). In co-cultures with immunosuppressive M2-polarized macrophages, combination treatment with atezolizumab and wild-type tiragolumab resulted in enhanced production of T cell cytokines IL-2 and TNF relative to single-agent treatment with atezolizumab (Fig. 6d). This effect was lost with deglycosylated tiragolumab, demonstrating the importance of FcγR engagement (Fig. 6d). Atezolizumab alone was sufficient to enhance T cell responses in co-cultures with pro-inflammatory M1-polarized macrophages, with no added tiragolumab benefit, suggesting that they are capable of supporting T cell responses independent of tiragolumab- and FcγR-mediated co-stimulation (Fig. 6e).

## Discussion

**Extended Data Fig. 10:**
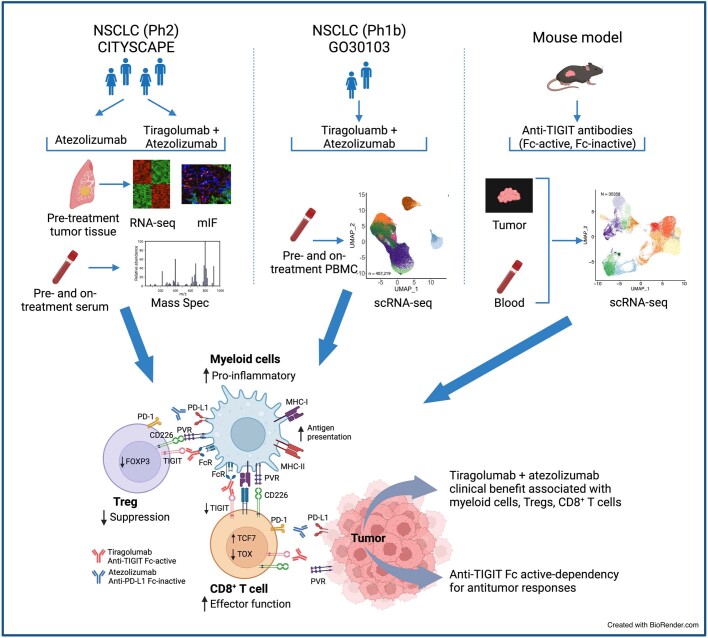
Graphic illustration showing the design of the current study. Top, To understand the mechanism(s) of response with tiragolumab in combination with atezolizumab, we leveraged samples collected from CITYSCAPE (left; NSCLC, Ph2) including tumour pretreatment samples for bulk RNA-seq and multiplex immunofluorescence (mIF), and pretreatment and on-treatment serum samples for Mass Spec, GO30103 (middle; NSCLC, Ph1b) including pretreatment and on-treatment peripheral blood mononuclear cells (PMBC) for single cell RNA-seq, and preclinical models (right). Bottom, Anti-TIGIT antibody, in a Fc dependent manner, remodels immunosuppressive tumour microenvironments by leveraging myeloid cells and Tregs, which was further enhanced with the addition of anti-PD-(L)1 antibody. Created with BioRender.com.

Checkpoint inhibitors enhance anti-tumour T cell responses, and clinical benefit is concentrated in patients in whom the tumours are rich in effector T cells and T-cell-driven inflammation^33^. By contrast, intratumoural macrophages, monocytes and T_reg_ cells are typically understood to suppress anti-tumour T cell responses and therefore resist the effects of checkpoint inhibitor treatment^34^. Notably, biomarker analysis of CITYSCAPE revealed that the improved therapeutic benefit of tiragolumab combination treatment was concentrated in tumours with high pretreatment levels of macrophages, monocytes and T_reg_ cells. Serum peptide and PBMC scRNA-seq analyses were also suggestive of an important role for macrophages and monocytes after tiragolumab + atezolizumab treatment. In preclinical models, anti-TIGIT monoclonal antibodies activated tumour and circulating myeloid cells through engagement of Fc receptors, and did so synergistically in combination with anti-PD-L1 antibodies. A second key effect of Fc-active anti-TIGIT antibodies was the induction of a memory-like gene program and downregulation of the gene program associated with exhausted effectors in tumour CD8^+^ T cells. Notably, anti-PD-L1 appeared to diminish this effect of anti-TIGIT treatment, at least in this context. These two effects were mechanistically linked, as these effects on CD8^+^ T cells were blunted when macrophages were depleted from tumours by treatment with anti-CSF-1R. Tumour T_reg_ cells also responded to Fc-active anti-TIGIT antibodies with downregulation of an immunosuppressive gene program, although this effect was independent of macrophages. The overall study design to investigate the mechanisms of action of anti-TIGIT monoclonal antibodies is summarized in Extended Data Fig. 10.

These clinical and preclinical data suggest that tiragolumab has a differentiated mechanism of action in which typically suppressive tumour myeloid cells potentiate rather than limit its activity. This mechanism appears to require engagement of activating Fc receptors, although it is probable that Fc-independent suppression of myeloid cell PVR signalling is also a contributing factor^15^. Fc-mediated activation of myeloid cells probably differentiates tiragolumab from checkpoint inhibitors that do not meaningfully engage Fc receptors, including antibodies targeting PD-L1, PD-1 and LAG-3. However, a recent report described similar myeloid-cell-activating effects of Fc-active ipilimumab and surrogate anti-CTLA-4 antibodies in preclinical models were also reported^35^, suggesting that this mechanism of action may be shared by Fc-enabled checkpoint inhibitors.

Myeloid cells exert a vast influence on the tumour microenvironment. The chemokines and cytokines that they produce are often determinative of the extent and composition of immune infiltration, and modulation of myeloid cells has been shown to influence T cell priming, activation, recruitment, survival and fate decisions^36–39^. It is important to note that there are substantial differences between mouse and human macrophage and Fc–Fc receptor biology^40,41^. Careful characterization of treatment effects in patients will be needed to further dissect the complex network of myeloid cell interactions, as well as the mechanistic contributions of Fc-active anti-TIGIT antibodies. To date, the importance of FcγR engagement to TIGIT antibodies has been uniquely controversial in the checkpoint inhibitor field, with antibodies in clinical development running the gamut from Fc-silenced to highly Fc-competent isotypes^6^. Our clinical and non-clinical findings now reveal a positive role for FcγR engagement in anti-TIGIT immunotherapy, suggesting that anti-TIGIT antibodies that are able to engage FcγR may deliver greater therapeutic benefit than those that cannot.

## Methods

### Study design, patient cohort and response assessment

Tissue and peripheral samples were obtained from patients enrolled in the open label, randomized phase 1b GO30103 (NCT02794571)^21^ and phase 2 CITYSCAPE (ClinicalTrials.gov: NCT03563716)^1^ trials. These trial protocols were approved by the institutional review board or ethics committee at each participating centre and complied with good clinical practice guidelines, the principles of the Declaration of Helsinki. Patients were required to have tissue sent to a central laboratory before study entry, and the samples were processed at the time of screening. Patients in the phase 1b study received escalating doses of tiragolumab alone or in combination with 1,200 mg atezolizumab every 3 weeks by intravenous dosing. CITYSCAPE evaluated atezolizumab with tiragolumab versus atezolizumab with placebo in chemotherapy-naive patients with locally advanced or metastatic NSCLC. Patients received either placebo plus 1,200 mg atezolizumab or 600 mg tiragolumab plus atezolizumab 1,200 mg every 3 weeks intravenously until disease progression or loss of clinical benefit. Protocol approval was obtained from independent ethics committees for each participating site for both studies and an independent data monitoring committee reviewed the safety data. Patient outcome was characterized as response (complete/partial response) or non-response (stable/progressive disease).

### Clinical tumour collection and bulk RNA-seq

Tumour biopsies at the baseline were collected from patients enrolled in the CITYSCAPE trial. Whole-transcriptome profiles were generated for *n* = 105 patients using TruSeq RNA Access technology (Illumina).

### Multiplex immunofluorescence

Multiplex immunofluorescence was performed on the Ventana Discovery ULTRA autostainer. After antigen retrieval with cell conditioning (CC1) solution (Ventana, 950-124), the samples were incubated with anti-FOXP3 rabbit monoclonal antibody SP97 (Abcam; ab99963), anti-pan-cytokeratin mouse monoclonal AE1/AE3 (Abcam, ab27988), anti-CD68 rabbit monoclonal SP251 (Spring Bioscience, M5510), anti-PD-L1 rabbit monoclonal SP263 (Ventana, 790-4905) and counterstained with DAPI (Thermo Fisher Scientific, D3106). Whole stained slide images were then aligned using UltiStacker software (Ultivue).

### Mass spectroscopy and ELISA

Serum samples were collected from patients enrolled in CITYSCAPE at C1D1 and C2D1. The samples were depleted of high-abundance proteins using the Agilent MARS human-14 multi-affinity removal column connected to the Dionex Ultimate 3000 RS pump (Thermo Fisher Scientific) according to the manufacturer’s instructions at Biognosys. The PQ500 panel had reference peptides (Biognosys) added to each sample.

Trypsinized serum was processed for hyper-reaction monitoring (HRM)/data independent acquisition (DIA) liquid chromatography–mass spectrometry measurements along with reference peptides using an HRM/DIA method, consisting of one full range MS1 scan and 29 MS2 segments, that was adopted from a previous study^42^.

HRM/DIA mass spectrometry data were analysed using Spectronaut software (Biognosys, v.14.10) and normalized using local regression normalization^43^. The mass spectrometry data were searched using SpectroMine (Biognosys, v.2.5), with a false-discovery rate on peptide and protein level set to 1%. Two separate spectral libraries were created from DDA data and directDIA data from HRM/DIA data. Low-quality protein levels were filtered on the basis of *Q*-values (cut-off, 0.01) and the batch-effect corrected using combat as described previously^44^. limma^45^ was used to test for differences in log-scaled protein levels. PQ500 assay panel data were used for clinical efficacy analysis. A composite was calculated at each timepoint (C1D1 and C2D1) by averaging scaled PQ500 values of all significantly increased proteins (MARCO, CAMP, CD163, CSF-1R, CD5L, NGAL (*LCN2*), GAPR1, APOC1, APOC2, APOC3 and APOC4).

The human CD163 immunoassay from R&D systems (DC1630) was qualified in procured human serum samples and then used to measure soluble CD163 from patient serum samples in duplicate.

### PBMC sample collection, RNA-seq library construction and sequencing

PBMCs were collected from patients enrolled in the phase 1b NSCLC study of tiragolumab plus atezolizumab (GO30103). A total of 16 patients had available samples from C1D1, C1D15 (2 weeks after treatment), C2D1 (3 weeks after treatment) and C4D1 (9 weeks after treatment). Frozen PBMCs were thawed, washed twice in RPMI 2% FCS, treated with the ACK lysis buffer (Lonza) to remove red blood cells (RBCs) and briefly incubated with DAPI. In total, 300,000 cells were then sorted on a DAPI-negative gate, stained for 30 min at room temperature with a custom panel of 139 Total-Seq-C antibodies (BioLegend)^46^ and washed three times using the HT1000 laminar wash system (Curiox) at Immunai. Cells were then counted using the Cellaca MX High-throughput Automated Cell Counter (Nexcelom), pooled from five samples, and loaded onto the 10x Chromium Next GEM Chip G Kit using a superloading strategy. TCR CDR3 sequences were enriched using human V(D)J T cell enrichment. Libraries were prepared according to the manufacturer’s protocol (10x Genomics) and sequenced on the NovaSeq 6000 System using the S4 2×150 kit (Illumina).

### Mice

C57BL/6J, BALB/c and FcγR-knockout mice were purchased from the Jackson Laboratory. All mice were housed and maintained at Genentech in accordance with American Association of Laboratory Animal Care guidelines. Mice were housed in individually ventilated cages within animal rooms maintained under a 14 h–10 h light–dark cycle. Animal rooms were temperature and humidity controlled, at 68–79 °F (20.0–26.1 °C) and 30–70%, respectively, with 10 to 15 room air exchanges per hour. Any mouse with a tumour larger than 2,000 mm^5^ was euthanized according to our guidelines from the Institutional Animal Care and Use Committee. All experimental animal studies were conducted under the approval of the Institutional Animal Care and Use Committees of Genentech Lab Animal Research and were performed in an Association for the Assessment and Accreditation of Laboratory Animal Care-accredited facility.

### Mouse surrogate therapeutic antibodies

A ligand-blocking anti-TIGIT antibody, clone 10A7, was generated as previously described^15^ and cloned onto mouse IgG2a, IgG2b and Fc-inert IgG2a-LALAPG backbones. The monoclonal mouse PD-L1 antibody 6E11 was generated as previously described and cloned into mouse IgG2a-LALAPG Fc effectorless backbone^47^.

### In vivo mouse tumour models

The CT26 mouse colon carcinoma cell line was obtained from American Type Culture Collection. The EO771 cell line was obtained from CH3 Biosystems (SKU940001). Cells were cultured in Roswell Park Memorial Institute (RPMI) 1640 medium containing 2 mM l-glutamine and 10% fetal bovine serum (HyClone). Cells were tested to be mycoplasma free before inoculation to mice. Cells in log-phase growth were centrifuged, washed once with Hank’s balanced salt solution (HBSS), counted and resuspended in 50% HBSS and 50% Matrigel (BD Biosciences). A total of 1 × 10^5^ CT26 cells was inoculated subcutaneously into the right unilateral flank of each mouse. For EO771 tumour studies, 1 × 10^5^ EO771 cells were injected into the fifth mammary fat pad of age-matched 6–8-week-old C57BL/6 female mice. After approximately 10–12 days, mice bearing tumours of 150–200 mm^3^ were randomized into treatment groups on the basis of tumour size and treated with anti-mouse PD-L1 (6E11, isotype IgG2a LALAPG, 10 mg per kg), anti-mouse TIGIT (10A7, isotypes IgG2a, mIgG2b, and IgG2a LALAPG, 10 mg per kg), anti-mouse CSF-1R (Bioexcell, BP0213, 30 mg per kg) and/or anti-gp120 control antibodies (IgG2a isotype, to total 35 mg per kg overall antibody dosing). For EO771 studies, anti-gp120 control antibodies (isotype IgG2a), anti-mouse PD-L1 (6E11, isotype IgG2a LALAPG) and anti-mouse TIGIT (10A7, isotype IgG2a or isotype IgG2a LALAPG) were used at 10 mg per kg. Anti-mouse PD-L1 and anti-mouse TIGIT antibodies were administered intravenously for the first dose and subsequently administered intraperitoneally.

In tumour growth inhibition and macrophage-depletion experiments, antibodies were administered three times per week for 2 weeks; the first dose was administered intravenously and all subsequent doses were administered by intraperitoneal injection. Animals were continuously monitored, and mice were euthanized by asphyxiation when any of the following end points were met: study termination, tumour burden ≥2,000 mm^3^, tumour ulceration, body weight loss of ≥20% or moribund appearance. Tumour burden was measured using callipers, and tumour volumes were calculated using the modified ellipsoid formula 1/2 × (length × width^2^). In scRNA-seq experiments, antibodies were administered once intravenously. Then, 72 h after treatment, mice were euthanized by asphyxiation and CD45^+^ cells were collected for scRNA-seq analyses. In anti-CSF-1R macrophage-depletion experiments, CD45^+^ cells were collected at 72 h after antibody treatment for scRNA-seq analysis and were collected at day 7–20 after the first dose of antibody treatment for FACS analyses.

### Ex vivo flow cytometry analysis of mouse tumours

Tumours were minced and digested with collagenase/DNase, filtered and resuspended in single-cell suspension for FACS staining. Fluorophore-conjugated antibodies against the indicated surface markers were used to stain single-cell solutions of tumours and peripheral blood cells. Cell surface staining was performed after gp70 tetramer staining. Cells were incubated for 20 min on ice with LIVE/DEAD Fixable Aqua Dead Cell Staining Kit (Invitrogen) and antibodies against CD45, CD4, CD8, CD11B, CD11C, MHCII, LY6G, LY6C, F4/80, CD86, CD25 and TIM3. For intracellular staining, cells were first stained with surface markers, fixed, permeabilized and stained with antibodies against FOXP3, TOX, TCF1 or Ki-67. For cytokine staining, cells were stimulated with a cell-stimulation cocktail pLus protein transporter inhibitors (eBioscience) for 3–4 h, stained with surface antibodies, fixed, permeabilized and stained with antibodies against IFNγ and TNF. All antibodies were purchased from BD Biosciences, BioLegend or eBioscience, except anti-TOX (Miltenyi) and anti-TCF1 (Cell Signalling) antibodies. Stained cells were analysed using the BD FACSymphony A5 Cell Analyzer flow cytometer, and further data analysis was performed using FlowJo software.

### Mouse RNA-seq library preparation and sequencing

Blood and tumours were collected from mice and processed for single-cell suspension preparation by enzymatic dissociation and/or red blood cell lysis as needed. Cells within each tissue and treatment group were hash-tagged (BioLegend TotalSeq C), pooled from different mice and labelled with fluorescent anti-CD45 antibodies and a viability dye. Live CD45^+^ cells were sorted and cell numbers determined using the Vi-CELL XR cell counter (Beckman Coulter). A total of 20,000 CD45^+^ cells was processed according to the 10x Genomics’ protocol (CG000330_Chromium Next GEM Single Cell 5-v2 Cell Surface Protein UserGuide_RevA) to generate 5′ single-cell RNA-seq and hashed libraries. Both libraries were sequenced on the NovaSeq S4 sequencer (Illumina) with the specifications based the 10x Genomics recommendations and as follows: 5′ single-cell RNA-seq libraries were sequenced at 40,000 reads per cell and hashed libraries at 2,000 reads per cell (Abiosciences).

### Human leukocyte co-culture experiments

Human PBMCs were isolated from healthy donors positive for CMV. PBMCs were treated with cytokines to drive polarization/differentiation into macrophages according to methods described previously^48^. In brief, for M1 macrophages, monocytes were treated with GM-CSF 50 ng ml^−1^ for 3 days followed by IFNγ 50 ng ml^−1^ along with LPS 10 ng ml^−1^ for 48 h; for M2 macrophages, monocytes were treated with M-CSF 50 ng ml^−1^ for 3 days followed by IL-4 20 ng ml^−1^, TGF-β 50 ng ml^−1^ and IL-10 50 ng ml^−1^ for 48 h. M1 macrophages were high for CD86, CD68, HLA-DR but low for CD200R and CD163, whereas M2 macrophages were high for CD163 and CD200R and low for HLA-DR. Macrophages were pulsed with CMV peptide (Anaspec) and the CEFX Ultrastim peptide pool (JPT peptides) and bulk CD3^+^ T cells from the same donors were introduced at a ratio of 5:1 in the presence of atezolizumab, tiragolumab or deglycosylated tiragolumab as described. Clinical grade tiragolumab and atezolizumab were used at 100 µg ml^−1^. For Fc-dependency studies, a deglycosylated version of tiragolumab was used at 100 µg ml^−1^. All reagents were endotoxin free. Cytokine measurements of IL-2 and TNF were done from the supernatant using the Luminex platform.

### Quantification and statistical analysis

#### Gene expression analysis of patient tumour bulk RNA-seq

All transcriptome profiles were generated using TruSeq RNA Access technology (Illumina). Alignment of RNA-seq reads to ribosomal RNA sequences was performed to remove ribosomal reads. The NCI build 38 human reference genome was then used to align the remaining reads using GSNAP v.2013-10-10, with a maximum of two mismatches per 75 base sequence (parameters: -M 2 -n 10 -B 2 -I 1 -N 1 -w 200000 -E 1 --pairmax-rna=200000 --clip-overlap) allowed^49^. Transcript annotation was based on the Ensembl genes database (release 77). To quantify gene expression levels, the number of reads mapped to the exons of each RefSeq gene was calculated in a strand-specific manner using the functionality provided by the R package Genomic Alignments (Bioconductor)^50^.

#### Public scRNA-seq processing and myeloid cell signatures

The scRNA-seq dataset for human lung tumours reported previously^51^ was obtained as .loom files from E-MTAB-6149 and is also available in the Laboratory for Functional Epigenetics^52^. Data were converted to a Seurat object and analysed using the Seurat R package (v.3.2.2) according to the standard workflow (Seurat)^53^. Myeloid cells were retrieved and analysed to define cell subtypes. As reported previously, cells were removed if there were either <201 unique molecular identifiers (UMIs), >6,000 or <101 expressed genes, or >10% UMIs derived from mitochondrial genome. The filtered gene expression matrix was normalized using the NormalizeData function with the default parameters. We then scaled the data and regressed out the effects of variation of UMI counts and percentage mitochondrial contents (ScaleData). Principal component analysis was performed on the scaled data cut to the top 2,000 variable genes defined by FindVariableFeatures with the default parameters. To integrate different samples, the harmony (v.1.0) package^54^ was used and the top 20 principal components (PCs) were used as input for the RunHarmony function with the default parameters. Cell clusters were defined using FindClusters using a resolution of 0.5 and annotated using canonical marker genes that were curated previously^55^.

Gene signatures were either derived from these NSCLC scRNA-seq datasets^51,56^ or have been described previously^57,58^. To derive the TAM signatures, markers for each myeloid cluster were defined by comparing cells in a particular myeloid cell cluster to every other cluster in a pairwise manner. To guarantee myeloid-specific expression of markers, we retained only marker genes that were not expressed by non-myeloid cells in an independent dataset^56^, including stromal, tumour and non-myeloid immune cells. We grouped three previously described macrophage populations^55^ characterized by their immunosuppressive characteristics as TAMs. We combined the signature genes from each defined macrophage cluster and derived the resultant signature (*MARCO*, *ACP5*, *VSIG4*, *MRC1*, *MSR1*, *MCEMP1*, *CYP27A1*, *OLR1*, *GRN*, *GLIPR2*, *ARRDC4*, *C1QC*, *APOE*, *FOLR2*, *CTSD* and *SPP1*).

#### Preprocessing of human PBMC scRNA-seq data

The scRNA-seq reads were aligned to the human transcriptome (GRCh38) and UMI counts were quantified to generate a gene–barcode matrix using the Cell Ranger pipeline (10x Genomics, v.5.0.1). CITE-seq antibody expression matrices were generated using the Cell Ranger pipeline (10x Genomics, v.5.0.1). TCR reads were aligned to the GRCh38 reference genome and consensus TCR annotation was performed using the Cell Ranger vdj pipeline (10x Genomics, v.5.0.1). To assign cells to their respective samples of origin, cells were demultiplexed with a modified HTOdemux function from the Seurat package, whereby the negative cluster was defined by minimal non-zero expression.

#### Cluster analysis of human PBMC immune cells

The preprocessed gene expression matrix generated by the Cell Ranger pipeline was imported into Seurat (v.3.2.2) for downstream analysis. As a quality-control step, genes that were expressed in less than ten cells were removed and cells were filtered on the basis of the number of detected genes, the number of detected UMIs, house-keeping gene expression and the percentage of mitochondrial gene expression. Cells that expressed less than ten house-keeping genes were removed. For UMIs, detected genes and mitochondrial gene expression, cut-offs were defined as the more conservative value between a hard predefined cut-off (UMIs: lower, 1,000; upper, 20,000; genes: lower, 200; upper, 5,000; mitochondrial gene expression, 10%) and a dataset-specific cut-off computed using interquartile ranges. Furthermore, RBC and platelet contaminants were removed using automated filtering algorithms. The filtered gene expression matrix (17,804 genes × 406,296 cells) was normalized using the NormalizeData function (normalization.method = “LogNormalize” and scale.factor = 10,000). Surface proteins were normalized using the centred-log ratio method. Variable genes were identified using the FindVariableFeatures function with the default parameters. Before dimensionality reduction, the data were scaled, and the effects of variation in UMI counts and percentage mitochondrial contents were regressed out (the ScaleData function). Principal component analysis was then performed on the scaled data cut to the variable genes. Batch effects were mitigated using the Harmony (v.1.0) package^54^. Shared nearest neighbours were computed, and cells were then clustered using graph community clustering methods. A UMAP was generated using the RunUMAP function. Cells were annotated using a cell type classifier taking into account RNA, surface proteins and TCR sequences, and further validated and refined by using immunai’s curated in-house signatures. Multi-omic data were further used to remove low-quality cells and previously undetected doublets (for example, cells that express both CD8 and CD4 protein tags, and cells that express both a high B cell signature and have a detected TCR).

#### Identification of proliferating cells in human PBMCs

To identify proliferating cells from the scRNA-seq data, cell proliferation scores at the S and G2M phases were calculated using the CellCycleScoring function from Seurat. Proliferating cells were called based on G2M phase scores of ≥0.22 or S phase scores of ≥0.22.

#### Pseudo-bulk differential gene expression analysis of human PBMCs

Differential gene expression (DEG) tests were performed by pseudo-bulk analysis, in which gene counts were aggregated (summed) for each sample and cell type. Samples per cell type that had less than 10 cells were removed. Differential expression analysis was performed using the limma-voom R package (v.3.44.3)^45^ for each cell type independently. Patient ID was added as a covariate to the design formulae to consider the paired design. Patients without matching pre- and on-treatment samples were removed. The moderated *t*-statistics from limma DEG tests were used as a preranked gene list input for pathway enrichment analysis, which was performed using the fgsea R package (v.1.14.0)^59^. In this analysis, we used the Hallmark gene set collected from MSigDB (v.7.2).

#### Preprocessing of mouse scRNA-seq data

The gene expression FASTQ files were aligned to the mouse transcriptome (mm10) and UMI counts were quantified to generate a gene–barcode matrix using the Cell Ranger pipeline (10x Genomics, v.6.1.1). Antibody-derived tag (ADT) expression FASTQ files were generated using the Cell Ranger pipeline (10x Genomics, v.6.1.1). The exported gene expression and ADT expression matrices were imported into the Seurat package for downstream analysis. ADT data were normalized with centred log-ratio transformation and the HTODemux function was used to assign mouse of origin for each singlet cell, and annotate doublets and singlets.

#### Clustering analysis of mouse scRNA-seq data

Three batches of mouse scRNA-seq data were generated and analysed separately according to the standard Seurat workflow as described above. Throughout the analysis, we confirmed the absence of batch effects introduced by samples or other technical factors, and therefore did not perform batch-effect removal in our data. Cells were annotated by canonical marker genes and high-expression marker genes in the cluster compared with the other cells.

Specifically, for the scRNA-seq data generated from mice treated with isotype control, anti-TIGIT-LALAPG, anti-TIGIT-IgG2b and anti-TIGIT-IgG2a antibodies, singlets and negative cells were used for downstream analysis. Cells collected from the peripheral blood were kept using the following filtering: mitochondrial % counts <5%, 1,000 < UMI counts <15,000 and 500 < gene counts < 3,500, resulting in a total of 26,174 cells. The first 25 PCs and a resolution of 1 were used for dimensionality reduction and clustering, and clusters with similar marker gene expression were combined. Cells collected from the tumour were retained using the following filtering: mitochondrial % counts < 5%, 1,000 < UMI counts <25,000, and 500 < gene counts < 6,000. We first used the top 25 PCs for dimensionality reduction and clustered all cells at a resolution of 0.6 to define the broader myeloid cells (*n* = 5,352) and T/NK lymphocytes (*n* = 21,407). Further clustering analysis was performed on the myeloid cells (top 20 PCs and a resolution of 0.9) and T/NK lymphocytes (top 23 PCs and a resolution of 0.9).

For the scRNA-seq data generated from mice treated with isotype control, anti-PD-L1, anti-TIGIT-IgG2b, and anti-TIGIT-IgG2a, anti-PD-L1 + anti-TIGIT-IgG2b, and anti-PD-L1 + anti-TIGIT-IgG2a antibodies, singlets were used for downstream analysis. Cells collected from the peripheral blood were kept using the following filtering: mitochondrial % counts < 5%, 1,000 < UMI counts < 20,000, and 500 < gene counts < 4,500, resulting in a total of 55,368 cells. The first 25 PCs and a resolution of 1 were used for dimensionality reduction and clustering, and clusters with similar marker gene expression were combined. Cells collected from the tumour were retained using the following filtering: mitochondrial % counts < 5%, 1,000 < UMI counts < 25,000, and 500 < gene counts < 6,000. We first used the top 25 PCs for dimensionality reduction and clustered all cells at a resolution of 0.1 to define the broader myeloid cells (*n* = 4,261) and T/NK lymphocytes (*n* = 35,358). Further clustering analysis was performed on the myeloid cells (top 20 PCs and a resolution of 0.9) and T/NK lymphocytes (top 20 PCs and a resolution of 0.6).

For the scRNA-seq data generated from mice treated with isotype control, anti-PD-L1 + anti-TIGIT-IgG2a, and anti-PD-L1 + anti-TIGIT-IgG2a + anti-CSF-1R antibodies, singlets were used for downstream analysis. Cells collected from the tumour were retained using the following filtering: mitochondrial % counts < 5%, 1,000 < UMI counts < 25,000, and 500 < gene counts < 6,000. We first used the top 20 PCs for dimensionality reduction and clustered all cells at a resolution of 0.6 to define the broader myeloid cells (*n* = 3,734) and T/NK lymphocytes (*n* = 21,575). Further clustering analysis was performed on the myeloid cells (top 20 PCs and a resolution of 0.6) and T/NK lymphocytes (top 25 PCs and a resolution of 0.6).

#### Differential gene expression analysis of mouse scRNA-seq data

Differential gene expression analysis was performed using two-tailed Wilcoxon rank-sum tests implemented in Seurat. The FindMarkers function was used to define the DEGs between cells from each treatment group. Volcano plots and bubble plots were used to visualize genes that were differentially expressed in each treatment group.

#### Statistical analysis

Survival outcomes, OS and PFS were analysed using the Kaplan–Meier method. Univariate Cox regressions were implemented to estimate HRs and 95% CIs. Statistical details of experiments, the number of repeats performed and statistical tests used are shown in the figure legends and the Methods.

### Reporting summary

Further information on research design is available in the Nature Portfolio Reporting Summary linked to this article.

## Online content

Any methods, additional references, Nature Portfolio reporting summaries, source data, extended data, supplementary information, acknowledgements, peer review information; details of author contributions and competing interests; and statements of data and code availability are available at 10.1038/s41586-024-07121-9.

### Supplementary information

Supplementary Figures Supplementary Figs. 1–7.

Reporting Summary

Supplementary Table 1 Baseline characteristics in CITYSCAPE (ITT population and the BEP).

Supplementary Table 2 Population demographics in patients whose tumors have high vs low cell types. *P* values were derived using Fisher’s exact test.

### Source data

Source Data Fig. 2

Source Data Fig. 3

Source Data Fig. 4

Source Data Fig. 5

Source Data Fig. 6

Source Data Extended Data Fig. 3

Source Data Extended Data Fig. 4

Source Data Extended Data Fig. 5

Source Data Extended Data Fig. 6

Source Data Extended Data Fig. 7

Source Data Extended Data Fig. 8

Source Data Extended Data Fig. 9

## Data Availability

The sequencing data generated in this study is available from the Gene Expression Omnibus (GEO) repository under accession code GSE260816. Up-to-date details on Roche’s Global Policy on the Sharing of Clinical Information and how to request access to related clinical study documents are available online (https://go.roche.com/data_sharing). Anonymized records for individual patients across more than one data source external to Roche cannot, and should not, be linked due to a potential increase in risk of patient re-identification. Source data of preclinical study data are provided with this paper, and source data of clinical study data is available from the European Genome-Phenome Archive (EGA) under the accession code EGAS50000000251. To request access to such data, researchers can contact devsci-dac-d@gene.com. The data will be released to such requesters with necessary agreements to enforce terms such as security, patient privacy and consent of specified data use, consistent with evolving, applicable data protection laws. Source data are provided with this paper.

## Code availability

All packages used in this study are publicly available. This study does not report original codes.
