# Publisher Correction: Anti-TIGIT antibody improves PD-L1 blockade through myeloid and T_reg_ cells

**DOI:** 10.1038/s41586-024-07562-2

**Published:** 2024-05-30

**Authors:** Xiangnan Guan, Ruozhen Hu, Yoonha Choi, Shyam Srivats, Barzin Y. Nabet, John Silva, Lisa McGinnis, Robert Hendricks, Katherine Nutsch, Karl L. Banta, Ellen Duong, Alexis Dunkle, Patrick S. Chang, Chia-Jung Han, Stephanie Mittman, Nandini Molden, Pallavi Daggumati, Wendy Connolly, Melissa Johnson, Delvys Rodriguez Abreu, Byoung Chul Cho, Antoine Italiano, Ignacio Gil-Bazo, Enriqueta Felip, Ira Mellman, Sanjeev Mariathasan, David S. Shames, Raymond Meng, Eugene Y. Chiang, Robert J. Johnston, Namrata S. Patil

**Affiliations:** 1 Genentech Inc., South San Francisco, CA USA; 2 Sarah Cannon Research Institute/Tennessee Oncology, PLLC, Nashville, TN USA; 3 Hospital Universitario Insular de Gran Canaria, Las Palmas, Spain; 4 Yonsei Cancer Centre, Yonsei University College of Medicine, Seoul, South Korea; 5 Institut Bergonie CLCC Bordeaux, Bordeaux, France; 6 Faculty of Medicine, University of Bordeaux, Bordeaux, France; 7 Clínica Universidad de Navarra, CIMA Universidad de Navarra Pamplona, Pamplona, Spain; 8 Vall d’Hebron Institute of Oncology (VHIO), Barcelona, Spain

**Keywords:** Non-small-cell lung cancer, Biomarkers

Correction to: *Nature* 10.1038/s41586-024-07121-9 Published online 28 February 2024

This article was originally published under standard Springer Nature License (© The Author(s), under exclusive licence to Springer Nature Limited). It is now available as an open-access paper under a Creative Commons Attribution 4.0 International license, © The Author(s). The error has been corrected in the HTML and PDF versions of the article.

