# Author Correction: Anti-TIGIT antibody improves PD-L1 blockade through myeloid and T_reg_ cells

**DOI:** 10.1038/s41586-024-07956-2

**Published:** 2024-08-20

**Authors:** Xiangnan Guan, Ruozhen Hu, Yoonha Choi, Shyam Srivats, Barzin Y. Nabet, John Silva, Lisa McGinnis, Robert Hendricks, Katherine Nutsch, Karl L. Banta, Ellen Duong, Alexis Dunkle, Patrick S. Chang, Chia-Jung Han, Stephanie Mittman, Nandini Molden, Pallavi Daggumati, Wendy Connolly, Melissa Johnson, Delvys Rodriguez Abreu, Byoung Chul Cho, Antoine Italiano, Ignacio Gil-Bazo, Enriqueta Felip, Ira Mellman, Sanjeev Mariathasan, David S. Shames, Raymond Meng, Eugene Y. Chiang, Robert J. Johnston, Namrata S. Patil

**Affiliations:** 1 Genentech Inc., South San Francisco, CA USA; 2 Sarah Cannon Research Institute/Tennessee Oncology, PLLC, Nashville, TN USA; 3 Hospital Universitario Insular de Gran Canaria, Las Palmas, Spain; 4 Yonsei Cancer Centre, Yonsei University College of Medicine, Seoul, South Korea; 5 Institut Bergonie CLCC Bordeaux, Bordeaux, France; 6 Faculty of Medicine, University of Bordeaux, Bordeaux, France; 7 Clínica Universidad de Navarra, CIMA Universidad de Navarra Pamplona, Pamplona, Spain; 8 Vall d’Hebron Institute of Oncology (VHIO), Barcelona, Spain

**Keywords:** Non-small-cell lung cancer, Biomarkers

Correction to: *Nature* 10.1038/s41586-024-07121-9 Published online 28 February 2024

In the version of the article initially published, the Data availability section did not include the accession codes for the sequencing data generated in this study (available from the Gene Expression Omnibus (GEO) repository under accession code GSE260816) and the clinical study data (available from the European Genome-Phenome Archive (EGA) under the accession code EGAS50000000251). The HTML and PDF versions of the article have now been updated.

