# Supplementary material for: Anti-TIGIT antibody improves PD-L1 blockade through myeloid and Treg cells
Source: Nature. 2024 Feb 28;627(8004):646–55. doi: 10.1038/s41586-024-07121-9 (PMC11139643; doi:10.1038/s41586-024-07121-9)

**Anti-TIGIT antibody improves PD-L1 blockade via myeloid and Treg cells**

Xiangnan Guan^1,&^, Ruozhen Hu^1,&^, Yoonha Choi^1,†^, Shyam Srivats^1,†^, Barzin Y. Nabet^1^, John Silva^1^, Lisa McGinnis^1^, Robert Hendricks^1^, Katherine Nutsch^1^, Karl L. Banta^1^, Ellen Duong^1^, Alexis Dunkle^1^, Patrick S. Chang^1^, Chia-Jung Han^1^, Stephanie Mittman^1^, Nandini Molden^1^, Pallavi Daggumati^1^, Wendy Connolly^1^, Melissa Johnson^2^, Delvys Rodriguez Abreu^3^, Byoung Chul Cho^4^, Antoine Italiano^5^, Ignacio Gil Bazo^6^, Enriqueta Felip^7^, Ira Mellman^1^, Sanjeev Mariathasan^1^, David S. Shames^1^, Raymond Meng^1^, Eugene Y. Chiang^1^, Robert J. Johnston^1,*,§^, and Namrata S. Patil^1,*,§^

^1^Genentech Inc., South San Francisco, CA, USA

^2^Sarah Cannon Research Institute/Tennessee Oncology, PLLC, Nashville, TN, USA
 ^3^Hospital Universitario Insular de Gran Canaria, Las Palmas, Spain

^4^Yonsei Cancer Centre, Yonsei University College of Medicine, Seoul, South Korea

^5^Institut Bergonie CLCC Bordeaux, Bordeaux, France

^6^Clínica Universidad de Navarra, CIMA Universidad de Navarra Pamplona, Pamplona, Spain

^7^Vall d’Hebron Institute of Oncology (VHIO), Barcelona, Spain

^&^Equal contribution, ^†^Equal contribution, ^§^Equal contribution

*Correspondence: patil.namrata@gene.com; johnston.robert@gene.com

Supplemental Figure 1. Gating strategy for Fig. 5a


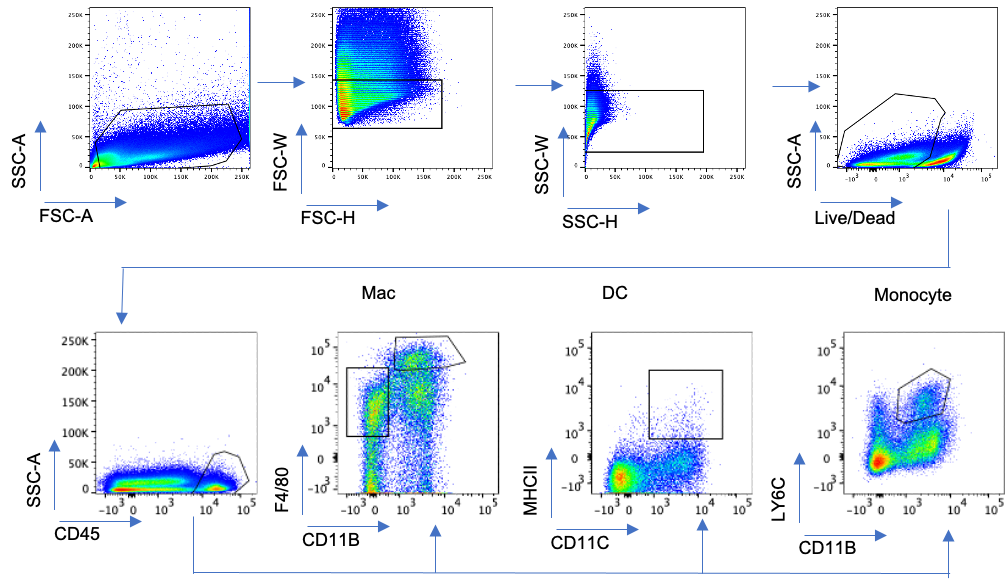


Supplemental Figure 2. Gating strategy for Fig. 5b, c


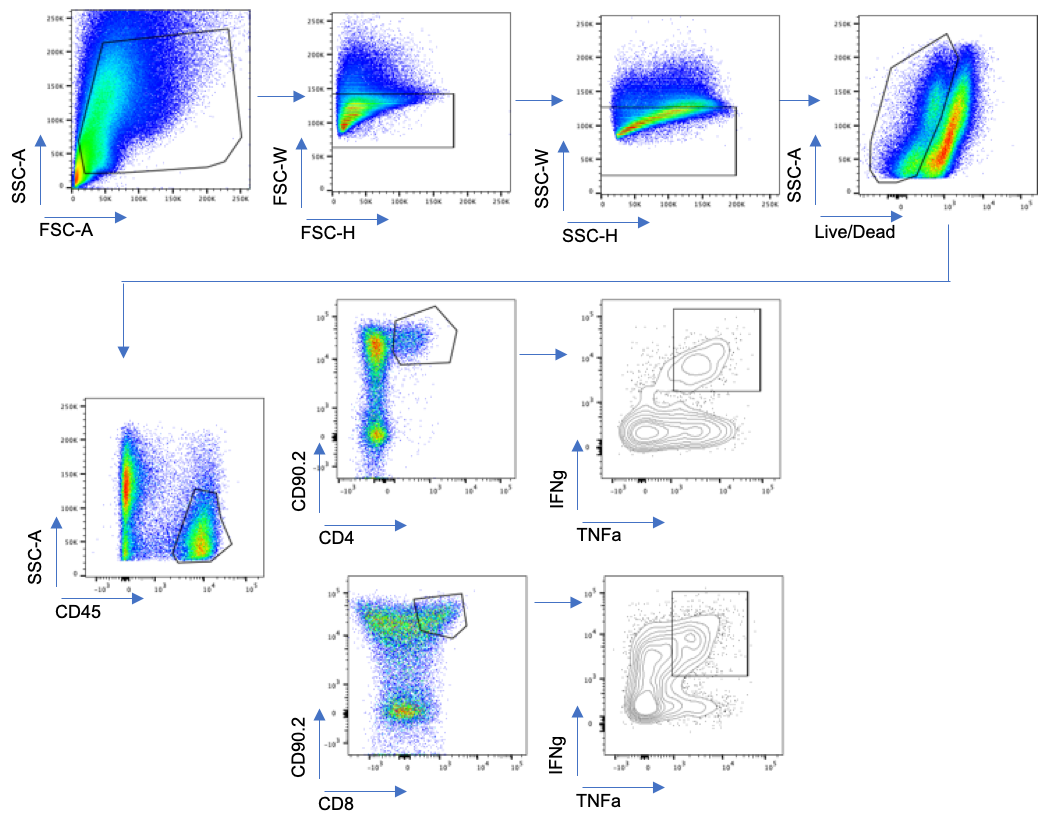


Supplemental Figure 3. Gating strategy for Fig. 5d-f & Extended Fig. 8d-e
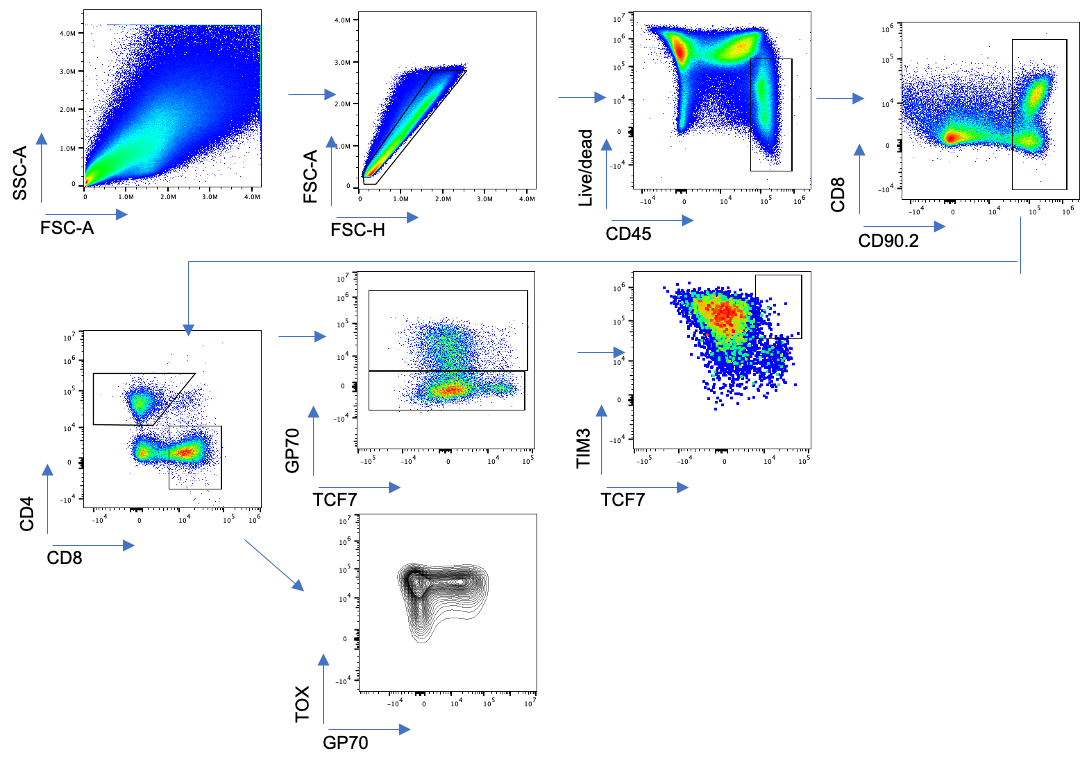


Supplemental Figure 4. Gating strategy for Fig. 6b


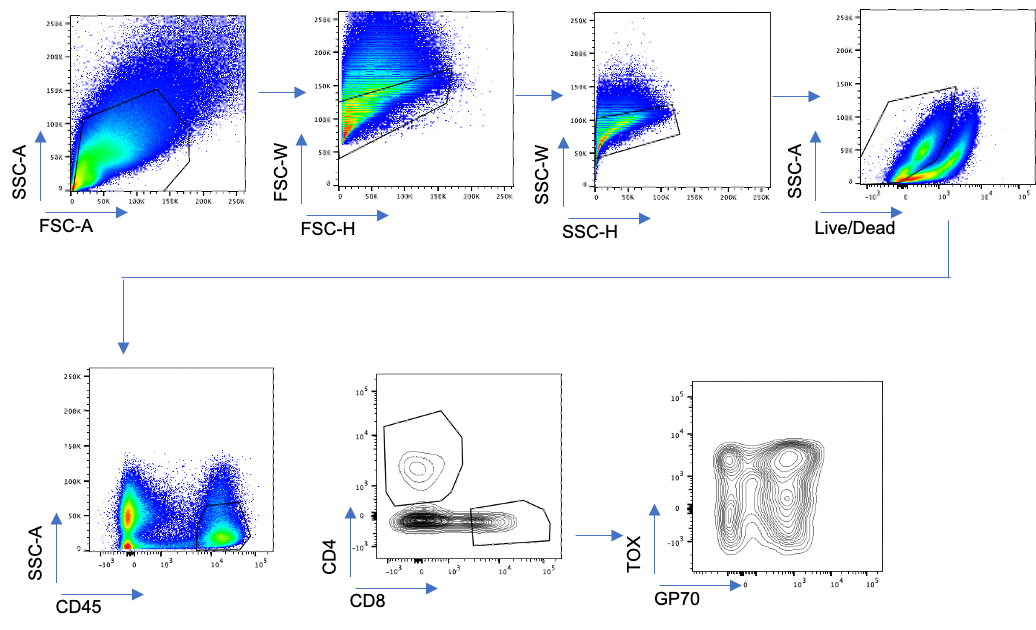


Supplemental Figure 5. Gating strategy for Extended Fig. 8a


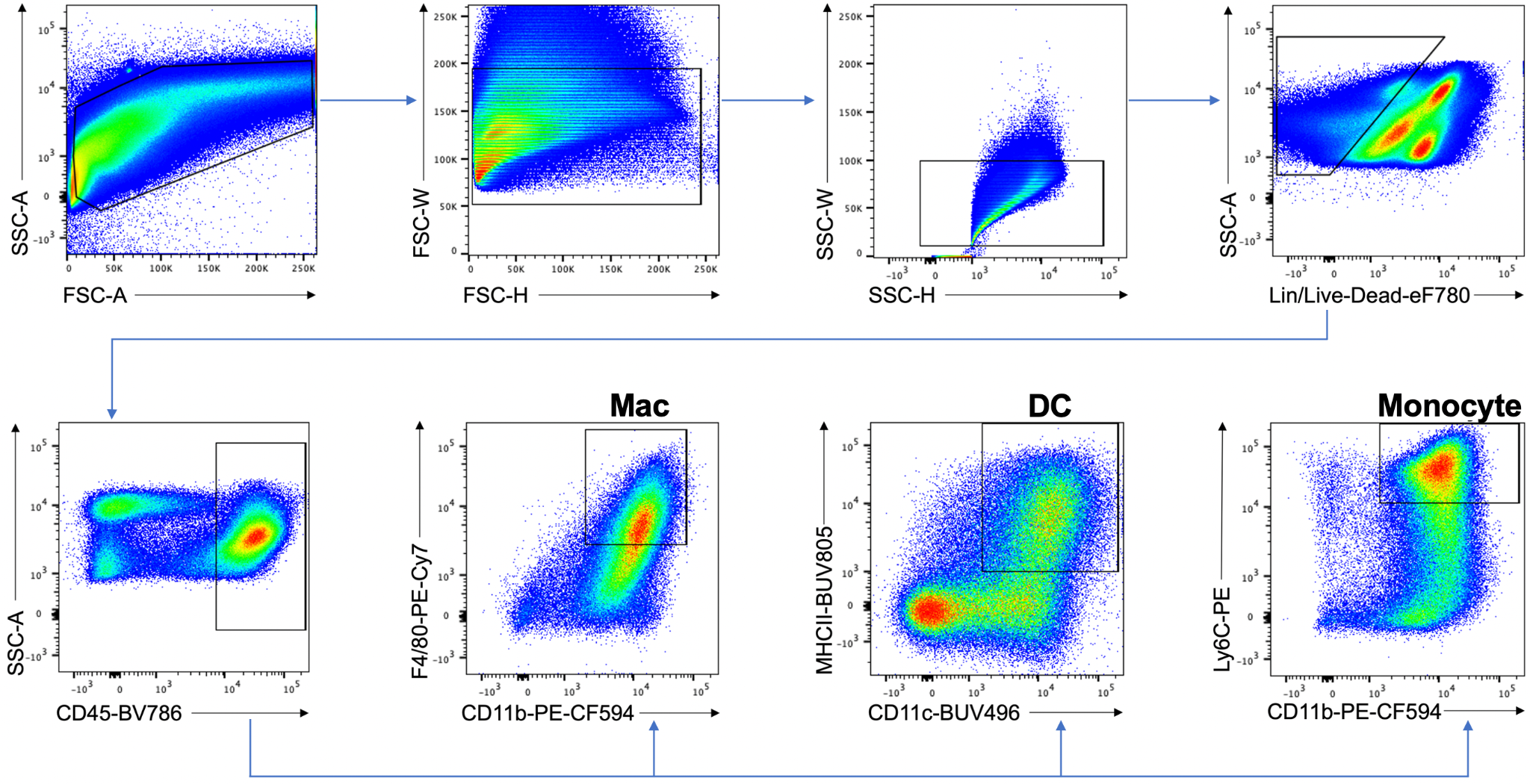


Supplemental Figure 6. Gating strategy for Extended Fig. 8b, c


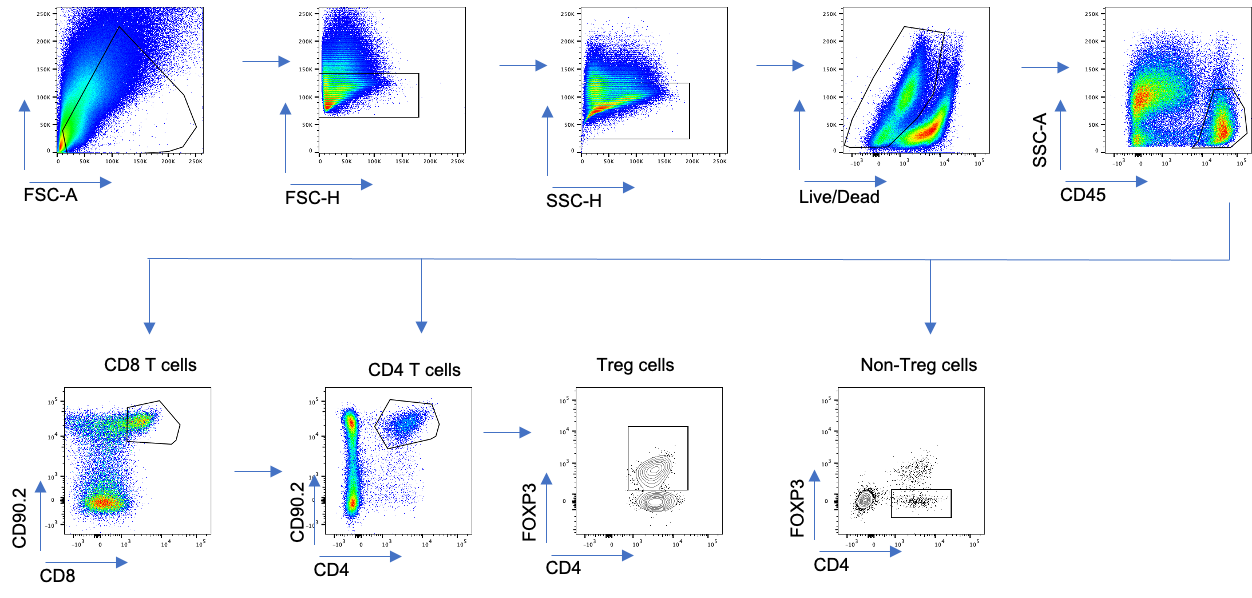


Supplemental Figure 7. Gating strategy for Extended Fig. 9a


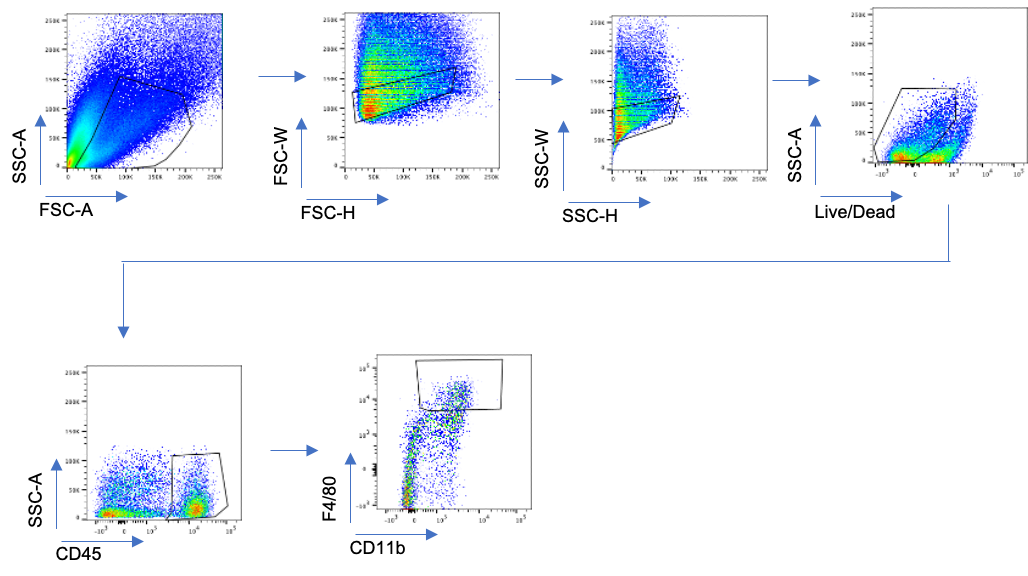

Supplement: Supplementary file 1 — Supplementary Figs. 1–7. [file 41586_2024_7121_MOESM1_ESM.docx]
