# Supplementary material for: Anti-TIGIT antibody improves PD-L1 blockade through myeloid and Treg cells
Source: Nature. 2024 Feb 28;627(8004):646–55. doi: 10.1038/s41586-024-07121-9 (PMC11139643; doi:10.1038/s41586-024-07121-9)
Supplement: Supplementary file 2 — Reporting Summary [file 41586_2024_7121_MOESM2_ESM.pdf]

nature portfolio

Corresponding author(s): Namrata S Patil

Last updated by author(s): Dec 11, 2023

Reporting Summary

Nature Portfolio wishes to improve the reproducibility of the work that we publish. This form provides structure for consistency and transparency in reporting. For further information on Nature Portfolio policies, see our Editorial Policies and the Editorial Policy Checklist.

Statistics

For all statistical analyses, confirm that the following items are present in the figure legend, table legend, main text, or Methods section.

n/a	Confirmed	
☐	☒	The exact sample size (*n*) for each experimental group/condition, given as a discrete number and unit of measurement
☐	☒	A statement on whether measurements were taken from distinct samples or whether the same sample was measured repeatedly
☐	☒	The statistical test(s) used AND whether they are one- or two-sided<br>*Only common tests should be described solely by name; describe more complex techniques in the Methods section.*
☒	☐	A description of all covariates tested
☐	☒	A description of any assumptions or corrections, such as tests of normality and adjustment for multiple comparisons
☐	☒	A full description of the statistical parameters including central tendency (e.g. means) or other basic estimates (e.g. regression coefficient) AND variation (e.g. standard deviation) or associated estimates of uncertainty (e.g. confidence intervals)
☐	☒	For null hypothesis testing, the test statistic (e.g. F, t, r) with confidence intervals, effect sizes, degrees of freedom and P value noted<br>*Give P values as exact values whenever suitable.*
☒	☐	For Bayesian analysis, information on the choice of priors and Markov chain Monte Carlo settings
☒	☐	For hierarchical and complex designs, identification of the appropriate level for tests and full reporting of outcomes
☒	☐	Estimates of effect sizes (e.g. Cohen's *d*, Pearson's *r*), indicating how they were calculated

Our web collection on statistics for biologists contains articles on many of the points above.

Software and code

Policy information about availability of computer code

Data collection	Flow cytometry data were collected using FACSymphony software (BD Biosciences).
Data analysis	No new code was developed for this study. All packages used in this study are publicly available as listed below.<br>Flow cytometry data were analyzed using FlowJo v10 and Prism v7<br>CellRanger (v5.0.1) for human single-cell data alignment and raw counts generation<br>CellRanger (v6.1.1) for mouse single-cell data alignment and raw counts generation<br>Spectronaut™ software (Biognosys, version 14.10) for mass spectrometric data<br>SpectroMine™ (Biognosys, version 2.5) for mass spectrometric data<br>GSNAP (v.2013-10-10) for bulk RNA-seq alignment.<br>R versiion 4.0.2 for all analysis done in the R environment<br>R package GenomicAlignments for gene expression quantification from bulk RNA-seq.<br>R package Seurat (v3.2.2) for single-cell processing and analysis.<br>R package Harmony (v1.0) for single-cell batch effect correction.<br>R package survminer (v0.4.8) for survival analysis.<br>R package survival (v.3.2.7) for survival analysis.<br>R package limma (v3.44.3) for differential gene expression analysis.<br>R package fgsea (v1.14.0) for pathway enrichment analysis.

For manuscripts utilizing custom algorithms or software that are central to the research but not yet described in published literature, software must be made available to editors and reviewers. We strongly encourage code deposition in a community repository (e.g. GitHub). See the Nature Portfolio guidelines for submitting code & software for further information.

Data

Policy information about availability of data

All manuscripts must include a data availability statement. This statement should provide the following information, where applicable:

- Accession codes, unique identifiers, or web links for publicly available datasets
- A description of any restrictions on data availability
- For clinical datasets or third party data, please ensure that the statement adheres to our policy

No new code was developed for this study.

GO30103 clinical data is obtained from Bendell,et al, 2020, AACR, https://aacrjournals.org/cancerres/article/80/16_Supplement/CT302/645050/Abstract-CT302-Phase-Ia-Ib-dose-escalation-study. CITYSCAPE clinical data is obtained from Cho, et al, 2022, Lancet Oncology, https://www.thelancet.com/journals/lanonc/article/PIIS1470-2045(22)00226-1/fulltext

Pathway enrichment analysis database (Molecular Signatures Database, MsigDB), https://www.gsea-msigdb.org/gsea/msigdb/

Research involving human participants, their data, or biological material

Policy information about studies with human participants or human data. See also policy information about sex, gender (identity/presentation), and sexual orientation and race, ethnicity and racism.

Reporting on sex and gender	Full description of the human research participants and characteristics is detailed in the following publicatios:<br>Bendell,et al, 2020, AACR, https://aacrjournals.org/cancerres/article/80/16_Supplement/CT302/645050/Abstract-CT302-Phase-Ia-Ib-dose-escalation-study.<br>Cho, et al, 2022, Lancet Oncology, https://www.thelancet.com/journals/lanonc/article/PIIS1470-2045(22)00226-1/fulltext
Reporting on race, ethnicity, or other socially relevant groupings	Full description of the human research participants and characteristics is detailed in the following publicatios:<br>Bendell,et al, 2020, AACR, https://aacrjournals.org/cancerres/article/80/16_Supplement/CT302/645050/Abstract-CT302-Phase-Ia-Ib-dose-escalation-study.<br>Cho, et al, 2022, Lancet Oncology, https://www.thelancet.com/journals/lanonc/article/PIIS1470-2045(22)00226-1/fulltext
Population characteristics	Patient characteristics are shown in Supplemental table 1 and the full description of the human research participants and characteristics is detailed in the following publications:<br><br>Cho, et al, 2022, Lancet Oncology, https://www.thelancet.com/journals/lanonc/article/PIIS1470-2045(22)00226-1/fulltext<br><br>Bendell,et al, 2020, AACR, https://aacrjournals.org/cancerres/article/80/16_Supplement/CT302/645050/Abstract-CT302-Phase-Ia-Ib-dose-escalation-study.
Recruitment	Full description of the human research participants and characteristics is detailed in the following publicatios:<br>Bendell,et al, 2020, AACR, https://aacrjournals.org/cancerres/article/80/16_Supplement/CT302/645050/Abstract-CT302-Phase-Ia-Ib-dose-escalation-study.<br>Cho, et al, 2022, Lancet Oncology, https://www.thelancet.com/journals/lanonc/article/PIIS1470-2045(22)00226-1/fulltext
Ethics oversight	The trial was conducted according to Good Clinical Practice and the Declaration of Helsinki. All patients provided written informed consent. Protocol approval was obtained from independent review boards or ethics committees at each site.

Note that full information on the approval of the study protocol must also be provided in the manuscript.

Field-specific reporting

Please select the one below that is the best fit for your research. If you are not sure, read the appropriate sections before making your selection.

☒ Life sciences ☐ Behavioural & social sciences ☐ Ecological, evolutionary & environmental sciences

For a reference copy of the document with all sections, see nature.com/documents/nr-reporting-summary-flat.pdf

Life sciences study design

All studies must disclose on these points even when the disclosure is negative.

Sample size	For the anlaysis of GO30103 and CITYSCAPE patient survival, the sample size was determined as described in Bendell,et al, 2020, AACR, https://aacrjournals.org/cancerres/article/80/16_Supplement/CT302/645050/Abstract-CT302-Phase-Ia-Ib-dose-escalation-study.<br>Cho, et al, 2022, Lancet Oncology, https://www.thelancet.com/journals/lanonc/article/PIIS1470-2045(22)00226-1/fulltext

	For all other analysis and experiments, no sample size calculation was conducted. For single cell CITE-seq patient cohort, we used patients from GO30103 who had available pre- and post-treatment PBMC. For preclinical experiments, a minimum of 5 mice per condition were used and for human donor ex vivo experiments, a minimum of 3 donors were used to minimize the impact of for donor to donor variability.
Data exclusions	For single cell CITE-seq data, we excluded cells with low and high number of detected transcripts, high mitochondria content, or high hemoglobin content.
Replication	For in vitro and in vivo experiment, we indicated in the figure legend or methods about the replication of each experiment.
Randomization	For preclinical experiments, randomization was as described in the methods. Randomization of GO30103 and CITYSCAPE is described in Bendell,et al, 2020, AACR, https://aacrjournals.org/cancerres/article/80/16_Supplement/CT302/645050/Abstract-CT302-Phase-Ia-Ib-dose-escalation-study.<br>Cho, et al, 2022, Lancet Oncology, https://www.thelancet.com/journals/lanonc/article/PIIS1470-2045(22)00226-1/fulltext
Blinding	The phase 2 clinical study CITYSCAPE was double blinded as described before. Full description of the human research participants and characteristics is detailed in the following publications:<br>Bendell,et al, 2020, AACR, https://aacrjournals.org/cancerres/article/80/16_Supplement/CT302/645050/Abstract-CT302-Phase-Ia-Ib-dose-escalation-study.<br>Cho, et al, 2022, Lancet Oncology, https://www.thelancet.com/journals/lanonc/article/PIIS1470-2045(22)00226-1/fulltext

Reporting for specific materials, systems and methods

We require information from authors about some types of materials, experimental systems and methods used in many studies. Here, indicate whether each material, system or method listed is relevant to your study. If you are not sure if a list item applies to your research, read the appropriate section before selecting a response.

Materials & experimental systems

n/a	Involved in the study	
☐	☒	Antibodies
☐	☒	Eukaryotic cell lines
☒	☐	Palaeontology and archaeology
☐	☒	Animals and other organisms
☐	☒	Clinical data
☒	☐	Dual use research of concern
☒	☐	Plants

Methods

n/a	Involved in the study	
☒	☐	ChIP-seq
☐	☒	Flow cytometry
☒	☐	MRI-based neuroimaging

Antibodies

Antibodies used	Antibodies used in flow cytometry:<br>anti-FOXP3 rabbit monoclonal antibody SP97 (Abcam; ab99963)<br>anti-pan-cytokeratin mouse monoclonal AE1/AE3 (Abcam, ab27988)<br>anti-CD68 rabbit monoclonal SP251 (Spring Bioscience, M5510)<br>anti-PD-L1 rabbit monoclonal SP263 (Ventana; 790-4905)<br>anti-mouse PD-L1 (Genentech, clone 6E11)<br>anti-mouse TIGIT (Genentech, clone 10A7)<br>anti-gp120 control antibody<br>Mouse BD Fc Block (BD Biosciences 553142)<br>AF700 Anti mouse CD8 (BD Biosciences 557959)<br>APC Anti mouse CD45 (BD Biosciences 559864)<br>anti-mouse CSF1R (Bioexcell, Cat# BP0213, 30 mg/kg)<br>Human TruStain FcX (Biolegend 422302)<br>Total-Seq-C antibodies (Biolegend)
Validation	The primary antibodies 6E11 and 10A7 isotypes were validated as reported before (Oh, Nature cancer 2020 and Johnston, Cancer Cell 2014 respectively). All the antibodies were validated by the manufacturer (Abcam, Spring Bioscience, Ventana, BD Biosciences, Biolegend).

Eukaryotic cell lines

Policy information about cell lines and Sex and Gender in Research

Cell line source(s)	The CT26 murine colon carcinoma cell line was obtained from American Type Culture Collection (Manassas, VA)<br>The EO77 murine colon carcinoma cell line was obtained from American Type Culture Collection (Manassas, VA)

Authentication	Cell lines were authenticated by Genentech.
Mycoplasma contamination	All cell lines were validated to be mycoplasma-free by PCR tests.
Commonly misidentified lines (See ICLAC register)	None

Animals and other research organisms

Policy information about studies involving animals; ARRIVE guidelines recommended for reporting animal research, and Sex and Gender in Research

Laboratory animals	Balb/c, C57BL/6J and FcgR knockout (KO) mice were purchased from the Jackson Laboratory. All experimental mice were female and 6-8 weeks old.
Wild animals	None
Reporting on sex	Female mice were used throughout the study.
Field-collected samples	None
Ethics oversight	All animal studies were approved by Genentech's Institutional Animal Care and Use Committee.

Note that full information on the approval of the study protocol must also be provided in the manuscript.

Clinical data

Policy information about clinical studies

All manuscripts should comply with the ICMJE guidelines for publication of clinical research and a completed CONSORT checklist must be included with all submissions.

Clinical trial registration	Data from GO30103 (NCT02794571) and CITYSCAPE (NCT03563716) detailed in the following publicatios:<br>Bendell,et al, 2020, AACR, https://aacrjournals.org/cancerres/article/80/16_Supplement/CT302/645050/Abstract-CT302-Phase-Ia-Ib-dose-escalation-study.<br>Cho, et al, 2022, Lancet Oncology, https://www.thelancet.com/journals/lanonc/article/PIIS1470-2045(22)00226-1/fulltext
Study protocol	The studies have reported and the full protocols are availabe on clinicaltrials.gov
Data collection	For full description of the data collection is described in Bendell,et al, 2020, AACR, https://aacrjournals.org/cancerres/article/80/16_Supplement/CT302/645050/Abstract-CT302-Phase-Ia-Ib-dose-escalation-study.<br>Cho, et al, 2022, Lancet Oncology, https://www.thelancet.com/journals/lanonc/article/PIIS1470-2045(22)00226-1/fulltext
Outcomes	For full description of the data collection and outcomes is described in Bendell,et al, 2020, AACR, https://aacrjournals.org/cancerres/article/80/16_Supplement/CT302/645050/Abstract-CT302-Phase-Ia-Ib-dose-escalation-study.<br>Cho, et al, 2022, Lancet Oncology, https://www.thelancet.com/journals/lanonc/article/PIIS1470-2045(22)00226-1/fulltext

Plants

Seed stocks	na
Novel plant genotypes	na
Authentication	na

Flow Cytometry

Plots

Confirm that:

☒ The axis labels state the marker and fluorochrome used (e.g. CD4-FITC).

☒ The axis scales are clearly visible. Include numbers along axes only for bottom left plot of group (a 'group' is an analysis of identical markers).

☒ All plots are contour plots with outliers or pseudocolor plots.

☒ A numerical value for number of cells or percentage (with statistics) is provided.

Methodology

Sample preparation	Flow sample preparation was detailed in the method.
Instrument	Data were collected on a BD LSRFortessa flow cytometer (BD Biosciences, San Jose, CA)
Software	Flow cytometry data were collected using FACSymphony software (BD Biosciences), and analyzed using FlowJo software.
Cell population abundance	Relevant cell population abundance is noted in each figure and is provided in the source data files for preclinical data.
Gating strategy	Gating strategy: cells were based in the following ways: singlets (FSC-A/FSC-H), live or dead cells (negative or negative dye staining), and then specific surface or intracellular markers.

☒ Tick this box to confirm that a figure exemplifying the gating strategy is provided in the Supplementary Information.
