# Supplementary figures and images for: Anti-TIGIT antibody improves PD-L1 blockade through myeloid and Treg cells
Source: Nature. 2024 Feb 28;627(8004):646–55. doi: 10.1038/s41586-024-07121-9 (PMC11139643; doi:10.1038/s41586-024-07121-9)

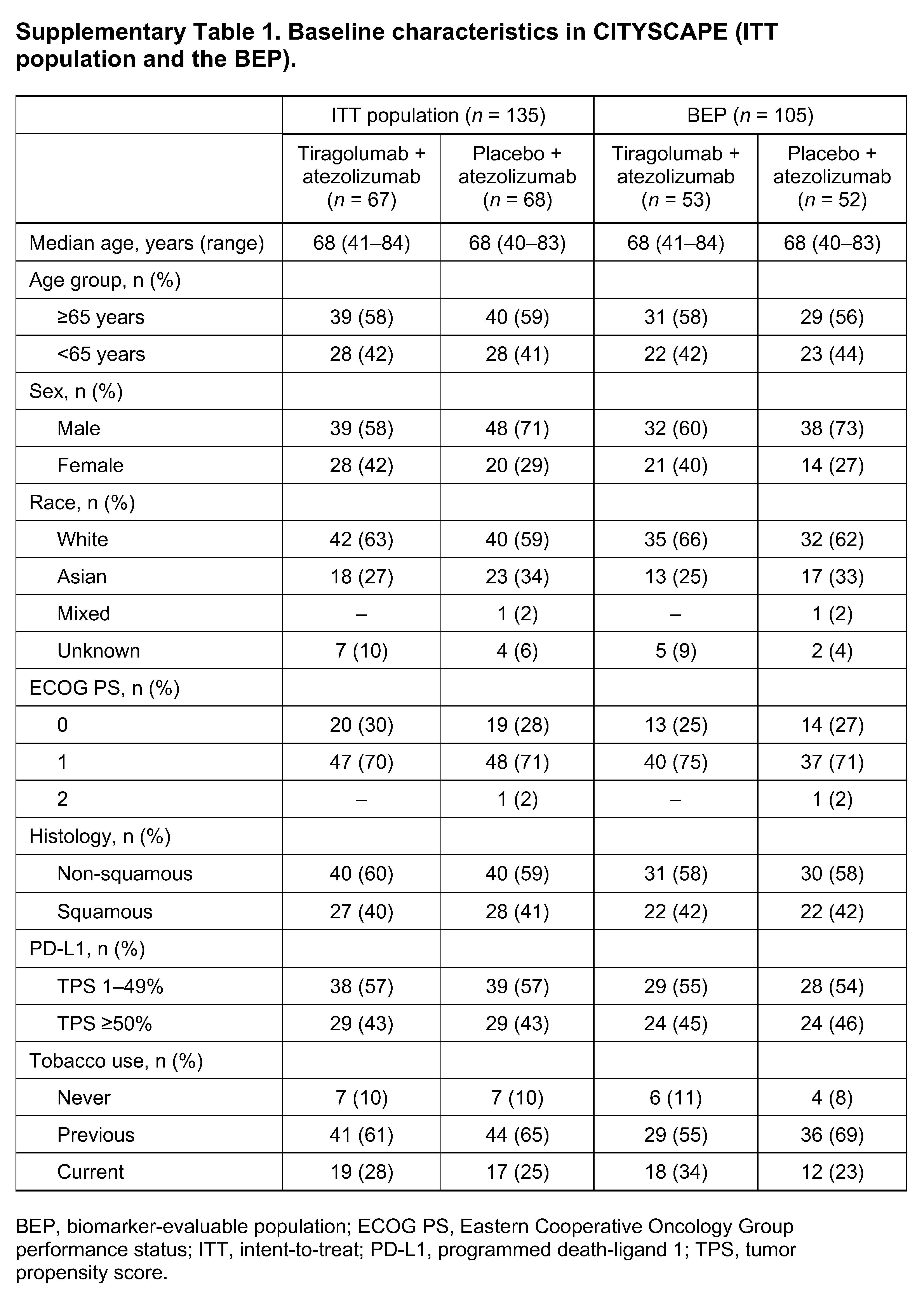

Supplement: Supplementary file 3 — Baseline characteristics in CITYSCAPE (ITT population and the BEP). [file 41586_2024_7121_MOESM3_ESM.jpg]

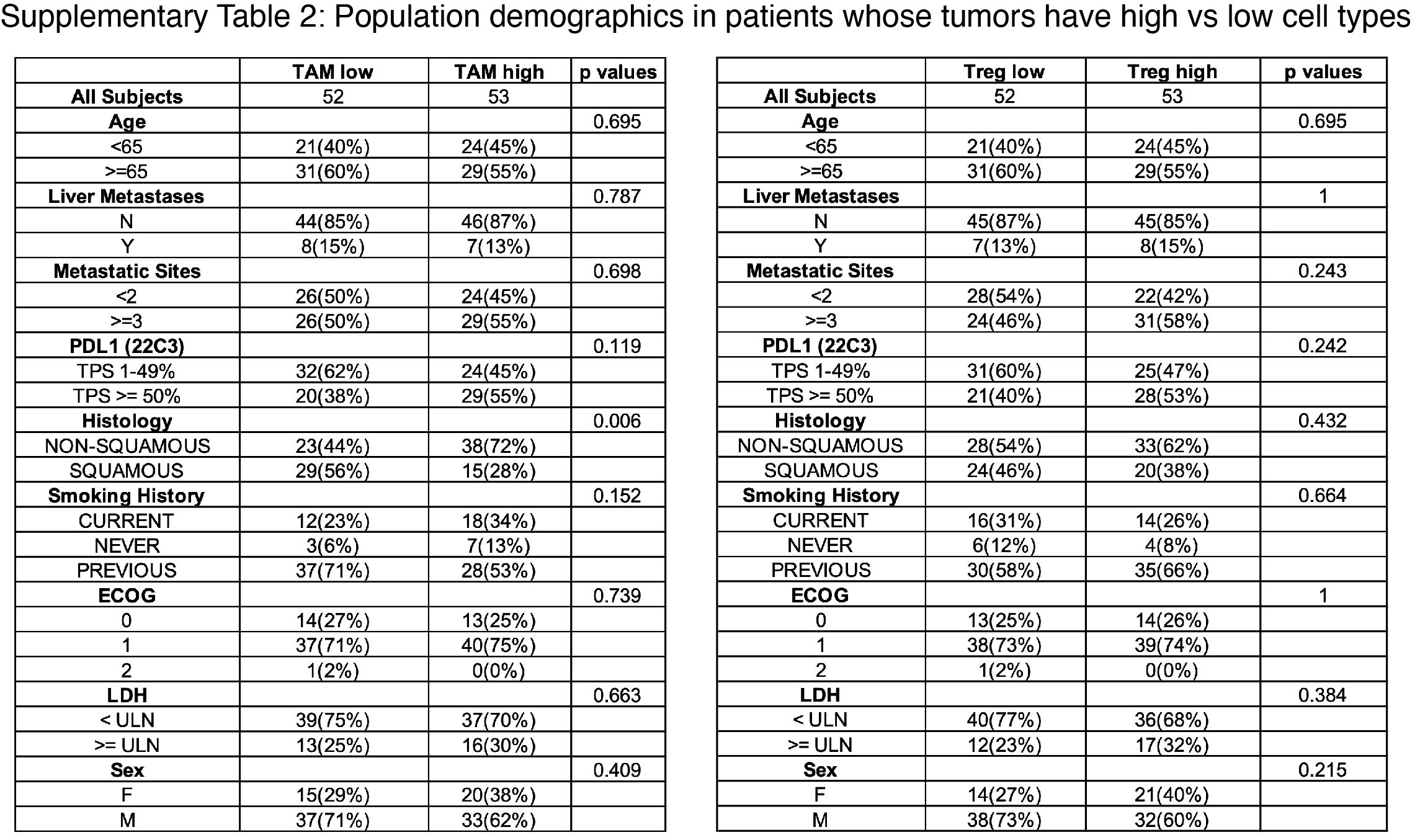

Supplement: Supplementary file 4 — Population demographics in patients whose tumors have high vs low cell types. P values were derived using Fisher’s exact test. [file 41586_2024_7121_MOESM4_ESM.jpg]
